# Elafin promotes tumour metastasis and attenuates the anti-metastatic effects of erlotinib via binding to EGFR in hepatocellular carcinoma

**DOI:** 10.1186/s13046-021-01904-y

**Published:** 2021-03-26

**Authors:** Chenwei Wang, Yadi Liao, Wei He, Hong Zhang, Dinglan Zuo, Wenwu Liu, Zhiwen Yang, Jiliang Qiu, Yichuan Yuan, Kai Li, Yuanping Zhang, Yongjin Wang, Yunxing Shi, Yuxiong Qiu, Song Gao, Yunfei Yuan, Binkui Li

**Affiliations:** 1 State Key Laboratory of Oncology in South China, Collaborative Innovation Center for Cancer Medicine, Sun Yat-sen University Cancer Center, Guangzhou, 510060 People’s Republic of China; 2 Department of Liver Surgery, Sun Yat-sen University Cancer Center, 651 Dongfeng Road East, Guangzhou, 510060 People’s Republic of China

**Keywords:** Elafin, Epidermal growth factor receptor, Metastasis, Erlotinib, Hepatocellular carcinoma

## Abstract

**Background:**

Elafin is a serine protease inhibitor critical for host defence. We previously reported that Elafin was associated with the recurrence of early-stage hepatocellular carcinoma (HCC) after surgery. However, the exact role of Elafin in HCC remains obscure.

**Methods:**

HCC tissue microarrays were used to investigate the correlation between Elafin expression and the prognosis of HCC patients. In vitro migration, invasion and wound healing assays and in vivo lung metastasis models were used to determine the role of Elafin in HCC metastasis. Mass spectrometry, co-immunoprecipitation, western blotting, and immunofluorescence staining assays were performed to uncover the mechanism of Elafin in HCC. Dual-luciferase reporter and chromatin immunoprecipitation assays were employed to observe the transcriptional regulation of Elafin.

**Results:**

Elafin expression was frequently increased in HCC tissues compared to normal tissues, and high Elafin expression in HCC tissues was correlated with aggressive tumour phenotypes and a poor prognosis in HCC patients. Elafin dramatically enhanced the metastasis of HCC cells both in vitro and in vivo by interacting with EGFR and activating EGFR/AKT signalling. Moreover, Elafin attenuated the suppressive effects of erlotinib on HCC metastasis. Besides, Elafin was transcriptionally regulated by Sp1 in HCC cells. Clinically, Elafin expression was positively correlated with Sp1, Vimentin, and EGFR signalling in both our HCC tissue microarrays and TCGA database analysis.

**Conclusions:**

Upregulation of Elafin by Sp1 enhanced HCC metastasis via EGFR/AKT pathway, and overexpression of Elafin attenuated the anti-metastatic effects of erlotinib, suggesting a valuable prognostic biomarker and therapeutic target for HCC.

**Supplementary Information:**

The online version contains supplementary material available at 10.1186/s13046-021-01904-y.

## Background

The high rates of recurrence and metastasis are the main factors contributing to death in hepatocellular carcinoma (HCC) patients, making HCC the fourth leading cause of cancer-associated death worldwide [1, 2]. Cancer metastasis is the result of a complex multi-step cell-biological process termed the invasion-metastasis cascade and is affected by tumour-intrinsic and tumour-extrinsic mechanisms [3]. Although great efforts have been made to investigate the metastasis of HCC, the detailed molecular mechanisms are still obscure.

Epidermal growth factor receptor (EGFR) has been reported to be frequently overexpressed in HCC, thereby promoting the tumorigenesis and progression of HCC [4]. EGFR gene amplification and elevated expression of EGFR ligands correlate with aggressive clinicopathological features in HCC [5]. In recent decades, the promising success of EGFR antagonists in non-small-cell lung cancer and colorectal cancer [6] and HCC cells and rat models [7] has set high expectations for HCC therapy. Frustratingly, several clinical phase II trials [8] and the SEARCH trial [9], the only phase III trial, have failed to show survival improvement with EGFR antagonists in advanced-stage HCC. Therefore, it is important to further understand the regulatory mechanisms of EGFR in HCC progression and to explore the application prospects of EGFR antagonists in HCC treatments.

We recently found that a three-CpG-based methylation signature based on peptidase inhibitor 3 (PI3), SCAN domain containing 3, and Src homology 3-domain growth factor receptor-bound 2-like interacting protein 1 could predict recurrence in patients with early-stage HCC after surgery [10]. PI3, also known as Elafin, belongs to the whey acidic protein family and is a serine protease inhibitor [11]. Numerous studies have focused on Elafin, which exhibits antimicrobial, anti-inflammatory, and wound healing functions by inhibiting the protease activity of human neutrophil elastase and proteinase 3 [12, 13]. Accumulating studies have shown that Elafin plays a complicated role in various malignancies [14]. Some studies have reported that Elafin promotes cell proliferation and induces chemotherapy resistance [15–17], while others have reported that Elafin mediates tumour-suppressive effects by inhibiting elastase and inducing apoptosis [18–20]. However, little is known about the role of Elafin in HCC.

In the present study, we identified Elafin as a key promoter of HCC metastasis. Briefly, Elafin could bind to EGFR and determine its critical roles by activating EGFR/AKT signalling. More importantly, Elafin attenuated the effects of erlotinib on suppressing HCC metastasis. These findings highlight that Elafin plays a critical role in HCC metastasis, suggesting that it may serve as a biomarker and therapeutic target for HCC.

## Materials and methods

### Human tissue specimens and HCC tissue microarray

Human HCC tissues and matched adjacent non-tumour liver tissues were obtained from patients who received curative surgery at the Sun Yat-sen Cancer Center (SYSUCC; Guangzhou, China). Two independent cohorts, including the training cohort (94 HCC specimens collected from December 2003 to July 2010) and the validation cohort (378 HCC specimens collected from January 2010 to May 2015) were used for survival analysis. The related clinicopathological features of the enrolled patients are presented in Table S1. All samples were obtained with the informed consent of the patients. This study complied with the standards of the 1975 Declaration of Helsinki and the experiments were approved by the Ethics Committee of Sun Yat-Sen University Cancer Center.

### Immunohistochemical staining (IHC)

After deparaffinized and blocked off the nonspecific antigen with goat serum (Zsbio, Beijing, China), HCC tissue microarrays were probed with anti-Elafin antibody, anti-Sp1 antibody, anti-Vimentin antibody, and anti-p-AKT antibody, as described in Table S4, overnight at 4 °C. Then, the microarrays were incubated with HRP anti-rabbit/mouse antibodies (Dako, Copenhagen, Denmark) for half an hour at 37 °C, and then the diaminobenzidine chromogen (Dako, Copenhagen, Denmark) was applied for reaction, followed by counterstaining with hematoxylin (Leagene, Beijing, China).

For IHC scoring, two experienced pathologists evaluated the staining intensity of specific markers, independently. The immunoreactivity for Elafin protein was scored using a semi-quantitative method by evaluating the number of positive tumor cells over the total number of tumor cells, and the IHC scores were assigned by using 5% increments (0, 5, 10%...100%), as described in previous studies [21, 22]. Based on the IHC scores, we then dichotomized these patients as negative group, 0–25%; weak group, 25–50%; moderate group, 50–75%; strong group, 75–100%. The high Elafin expression group is defined as patients with tumors of moderate or strong intensities, while the low Elafin expression group is defined as patients with tumors of negative or weak intensities [23, 24].

### Cell lines and cell culture

Cell lines and cell culture were performed as described previously [25]. Human HCC cell lines (PLC-8024, Huh7, Hep3B, MHCC-97H, and HepG2) and MIHA (the normal liver cell line) were purchased from the Shanghai Cell Bank of the Chinese Academy of Sciences (Shanghai, China) with STR (short tandem repeat) appraisal certificates. Cells were maintained in Dulbecco’s Modified Eagle medium (DMEM; ThermoFisher, USA) supplemented with 10% fetal bovine serum (FBS; Gibco, Califonia, USA) at 37 °C in 5% CO_2_.

### Co-immunoprecipitation (Co-IP) assay and mass spectrometry

The cells were lysed with RIPA (Fdbio Science, Hangzhou, China) lysis buffer supplemented with protease inhibitors, and the lysates were mixed with the corresponding conditioned medium of the cells. Fifty microlitres of the mixture was removed and used as input, and the remaining mixture was incubated with primary anti-EGFR or anti-Elafin or with anti-IgG as a negative control overnight, as described in Table S4, at 4 °C. Thereafter, an appropriate volume of Protein A/G PLUS-Agarose beads (sc-2003, Santa Cruz Biotechnology) was added to the mixture for conjugation for another 4 h at 4 °C according to the manufacturer’s protocol. After that, the beads were washed with RIPA buffer, and the precipitated proteins binding on the beads were collected. Finally, the beads were resuspended in electrophoresis loading buffer and boiled for western blotting assays with anti-EGFR or anti-Elafin antibodies. Mass spectrometry was conducted by Saizhe Science (Guangzhou, China) using PLC-8024 cells.

### Dimerization and internalization of EGFR

After starvation for 12 h, wild-type HCC cells were stimulated with 10 μg/ml recombinant Elafin (rElafin, R&D Systems, MN, USA) or 20 ng/ml EGF (236-EG, R&D Systems, MN, USA) or with BSA (Biofroxx, Germany) as the control for 30 min. After that, the cells were treated with the crosslinking reagent BS^3^ reagent (Thermo Fisher, MI, USA) at a concentration of 3 mM for 30 min at room temperature. Then, 40 μL 1 M Tris-HCl pH 7.5 was added for an additional 15 min to terminate the reaction. Finally, the cells were lysed and subjected to western blotting to assess EGFR dimerization. The dimers were represented by bands at approximately 300 KD, while EGFR monomers were represented by bands at 170 KD.

To assess the internalization of EGFR, after starvation for 12 h, the cells were stimulated with 10 μg/ml recombinant Elafin or 20 ng/ml EGF or BSA for 30 min. Then, the cells were subjected to immunofluorescence (IF) to assess the cell surface expression of EGFR.

### Elafin-EGFR docking

To further investigate the detailed binding mode of Elafin and EGFR, we docked Elafin to EGFR via the ClusPro online server (https://cluspro.bu.edu) with the default settings. Considering the similarity between Elafin and EGF, both of which are composed of ~ 50 residues and bind to the extracellular domain (residues 25–645) of EGFR, we selected the crystal structure of the EGFR/EGF complex (PDB ID: 1IVO) as a receptor for the docking procedure. The crystal structure of Elafin was derived from its complexed form (PDB ID: 1FLE). Among the resulting top 10 best binding modes, we chose the top one based on the analysis of interaction effects. To verify the predicted binding mode, we also repeated the docking through the Zdock online server (http://zdock.umassmed.edu), which yielded a result consistent with that of ClusPro.

### Luciferase reporter assay

Luciferase reporter plasmids, including full-length, different truncated, and mutant Elafin promoters, were constructed by GeneCopoeia (Rockville, USA). For the assay, PLC-8024 and Huh7 cells (5 × 10^4^) were seeded in 24-well plates under regular conditions and then co-transfected with Sp1 overexpression plasmids, or Sp1 siRNA or negative controls, together with Elafin promoters. After 48 h, the conditioned medium was collected, and the Gaussia luciferase (Gluc) and secreted alkaline phosphatase (SEAP) luciferase activities were measured consecutively using the Secrete-Pair™ Dual Luminescence Assay Kit (GeneCopoeia, Rockville, USA) according to the manufacturer’s instructions. The gluc activity was normalized to the SEAP activity, and each group was analysed in triplicate experiments.

### Chromatin Immunoprecipitation (ChIP) assay

ChIP assays were carried out in PLC-8024 and Huh7 cells with a ChIP kit (Cell Signaling Technology, Boston, USA) according to the manufacturer’s instruments. Briefly, 1% formaldehyde (Sigma-Aldrich, Germany) solution was added to induce PLC-8024 or Huh7 cell crosslinking followed by glycine solution to quench the reaction. Afterwards, the cells were lysed, and the nucleoprotein complexes were sonicated for 10 cycles of 10 s power-on and 20 s interval with an intensity of 200 W with the sonicate conductor (Qsonica, USA). Then, anti-Sp1 antibody or IgG, as described in Table S4, was added and incubated with the complexes overnight at 4 °C. The next day, Protein A/G magnetic beads were added to precipitate the indicated fragments for an addition 4 h at 4 °C. After extraction and purification of the indicated DNA, semiquantitative PCR was performed to identify the region interacting with the Elafin specific primers. The indicated primers were present in Table S5. The experiments were performed in triplicate and the amount of immunoprecipitated DNA was normalized to the input.

### In vivo lung metastasis model

To establish the lung metastasis model, 5- to 6-week old male nude mice (*n* = 12 per group) were injected with stable PLC-8024 or Huh7 cells (2 × 10^6^ cells suspended in 200 μL PBS) through the tail veins. Fifty days after injection, the mice were sacrificed, and the lungs were harvested for haematoxylin and eosin (H&E) staining, which was used to count the number of metastatic nodes in lung sections.

To assess the effects of erlotinib treatment, after injection with Huh7 stable Elafin overexpressing or vector cells (2 × 10^6)^ via the tail veins of the nude mice, the mice were treated with erlotinib (S1023, Selleck Chemicals) in PBS with 10% Captisol (HY-17031, MedChemExpress) at 80 mg/kg orally [26] or with vehicle (PBS with 10% Captisol) daily from the 4th to 7th week after injection. On day 50, the mice were sacrificed, and the lungs were collected. H&E staining was performed on paraffin-embedded lungs to identify the metastatic nodes in the lungs.

All BALB/c nude mice were purchased from the Medical Experimental Animal Center of Guangdong Province (China) and fed at the Animal Experimental Center of Sun Yat-Sen University. All animal research procedures were performed according to the Animal Care and Use Ethics Committee of Sun Yat-Sen University Cancer Center.

### Analysis of the Cancer genome atlas (TCGA) and gene expression omnibus (GEO) databases

The clinicopathological features, as well as data on the relative expression levels of Elafin, Sp1, and vimentin in the 371 HCC tissues, were downloaded from the TCGA-Liver Hepatocellular Carcinoma RNA sequencing dataset. The survival and expression data from another 221 HCC patients were previously deposited in the National Center for Biotechnology Information (NCBI) GEO dataset with accession number: GSE 14520.

### Statistical analysis

The experiments were repeated at least three times independently, and the measured data were represented as the mean ± SD. Binary variables were compared by using the Chi-squared test, and ordinal categorical variables were compared by the Kruskal-Wallis test. In the TCGA and GEO datasets, the optimal cutoff points for the expression of Elafin was determined by the maximally selected rank statistic from the “maxstat” R package for survival analysis. Survival curves were constructed using the Kaplan-Meier method and analysed by the log-rank test. Significant prognostic factors found by univariate analysis were entered into a multivariate analysis using the Cox proportional hazards model. The correlations between Elafin and vimentin or Sp1 or pAKT were assessed by Spearman correlation analysis. All analyses were two-sided, and differences with *P* values less than 0.05 were considered significant. Statistical analyses were performed using the R program (R version 3.5.0; R Foundation for Statistical Computing, Vienna, Austria) and GraphPad Prism 7.0 software (GraphPad, Inc., La Jolla, CA, USA).

Other experiments are described in the ‘Supplementary Data’.

## Results

### Elafin is upregulated in HCC tissues and is closely related to the prognosis of HCC patients

**Fig. 1:**
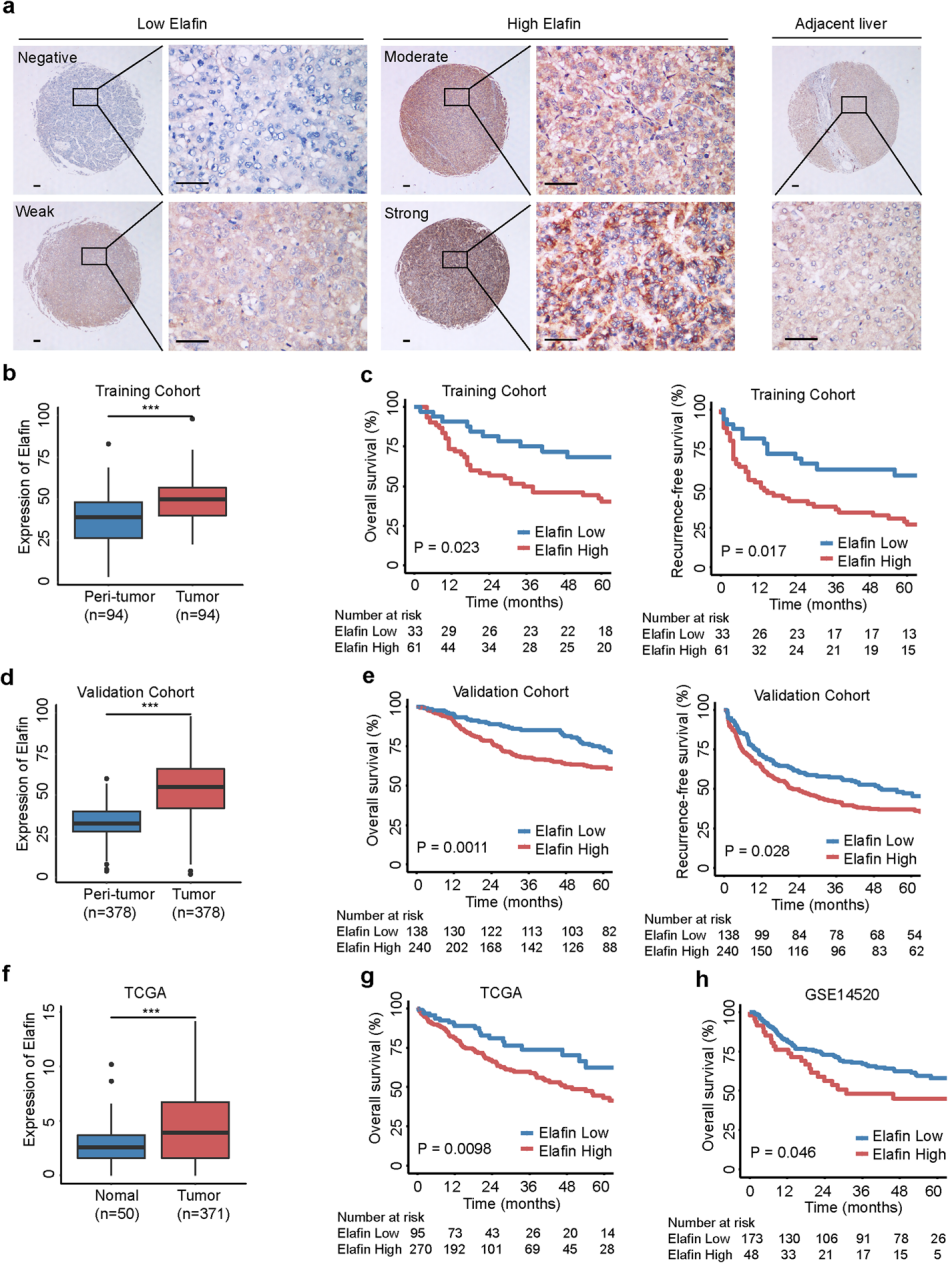
Elafin is up-regulated and correlates with poor prognosis in HCC. **a** The representative images of different levels of Elafin expression in HCC (left) and adjacent noncancerous liver tissues (right) detected by the immunohistochemical staining (IHC) are presented. Scale bar, 100 μm. **b** Quantification of Elafin expression in HCC and peri-tumor tissues according to IHC scores in SYSUCC training cohort (*n* = 94). Relative IHC scores are shown as mean ± SD. *** *P* < 0.001, based on the Wilcoxon matched pairs test. **c** Overall survival (OS) and Recurrence-free survival (RFS) curves with risk tables for patients with high and low Elafin expression in SYSUCC training cohort (*n* = 94). **d** Quantification of Elafin expression in HCC and peri-tumor tissues according to IHC scores in SYSUCC validation cohort (*n* = 378). Relative IHC scores are shown as mean ± SD. *** *P* < 0.001, based on the Wilcoxon matched pairs test. **e** OS and RFS curves with risk tables for patients with high and low Elafin expression in SYSUCC validation cohort (*n* = 378). **f** Relative of Elafin expression in HCC (*n* = 371) and normal tissues (*n* = 50) is determined based on TCGA dataset. *** *P* < 0.001. **g** OS curves with risk tables for patients with high and low Elafin expression based on TCGA HCC dataset (*n* = 371). **h** OS curves with risk tables for patients with high and low Elafin expression based on GEO database (GSE14520, *n* = 221)

We previously reported that Elafin was associated with the recurrence of early-stage HCC after surgery [10]. In the present study, we focused on PI3, which encodes the Elafin protein, for further investigation. The expression of Elafin was found to be significantly elevated in HCC tissues compared with paired adjacent liver tissues (Fig. S1A and Fig. 1a and b). HCC patients in the high Elafin expression group showed shorter overall survival (OS) and recurrence-free survival (RFS) than patients in the low expression group in the training cohort (Fig. 1c). Moreover, we verified these results in the validation cohort (Fig. 1d and e). Furthermore, high Elafin expression was significantly associated with high alpha-fetoprotein levels, cirrhosis, multiple tumours, lack of a tumour capsule, tumour thrombus, and adjacent organ invasion (all *P* < 0.05; Table S1). In the Cox proportional hazards regression analysis, high Elafin expression was an independent unfavourable prognostic factor for both OS and RFS (Table S2 and Table S3). Finally, the analysis of the TCGA and GEO datasets also verified our above findings (Fig. 1f, g, and h). These results suggest that elevated Elafin expression might correlate with aggressive clinicopathological characteristics and a poor prognosis in HCC patients.

### Silencing of Elafin suppresses the epithelial-mesenchymal transition (EMT) and metastasis of HCC cells in vitro and in vivo

**Fig. 2:**
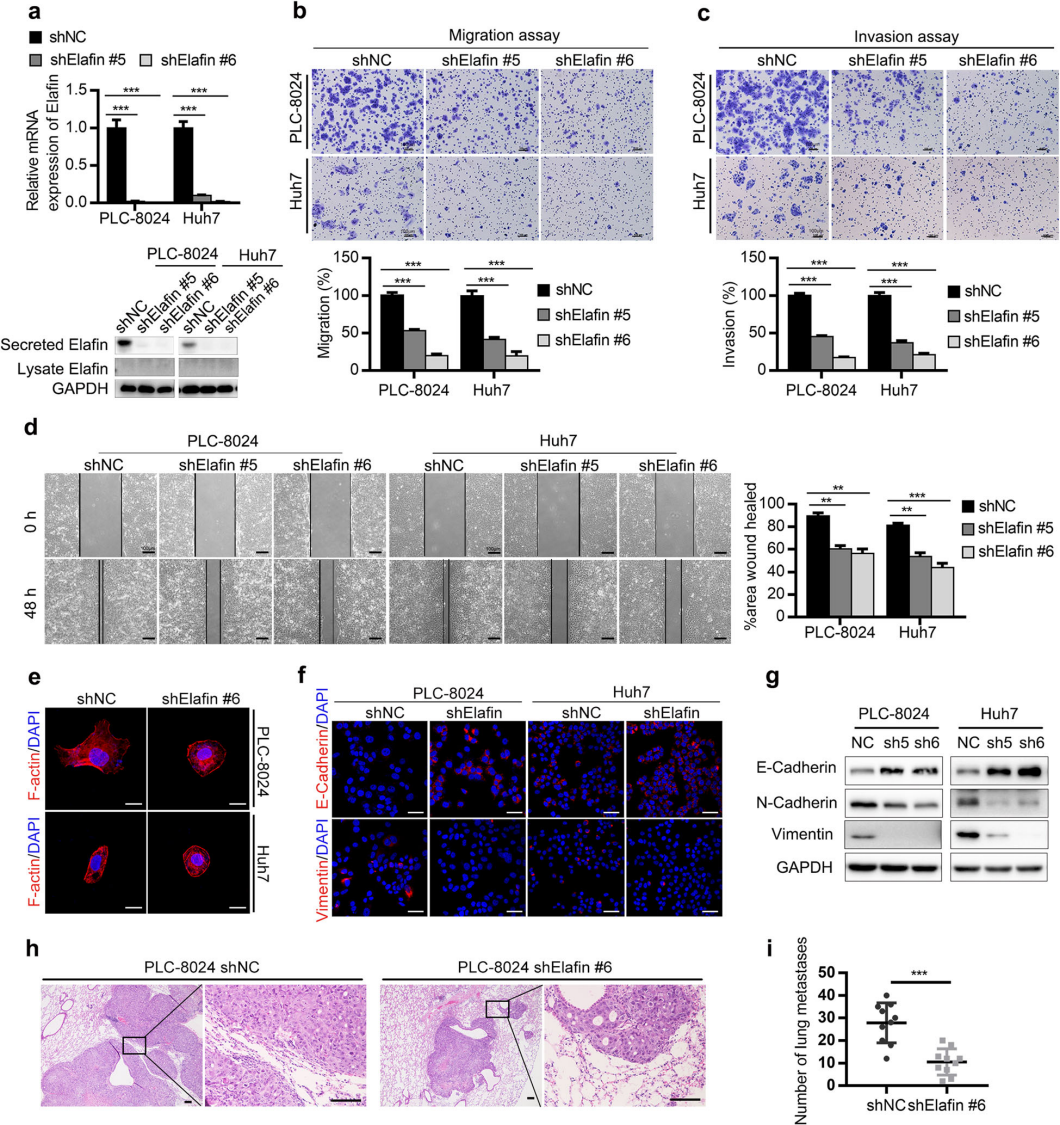
Knockdown of Elafin inhibits epithelial-mesenchymal transition (EMT) and metastasis of HCC cells in vitro and in vivo. **a** The efficiency of Elafin knockdown were measured by real-time PCR (top) and Western blotting (bottom) assays. *** *P* < 0.001. **b** Knockdown of Elafin resulted in suppressing migration of PLC-8024 and Huh7 cells. Scale bar, 100 μm. Statistical results are presented as mean ± SD (from triplicates), and significance is determined by Student *t* test (*** *P* < 0.001). **c** Knockdown of Elafin resulted in suppressing invasion of PLC-8024 and Huh7 cells. Scale bar, 100 μm. Statistical results are presented as mean ± SD (from triplicates) (*** *P* < 0.001). **d** Knockdown of Elafin impaired the scratch wound-healing ability of PLC-8024 and Huh7 cells. Scale bar, 100 μm. Statistical results at 48 h of scratch wound-healing assays are presented as mean ± SD (from triplicates) (** *P* < 0.01; *** *P* < 0.001). **e** Representative immunofluorescence images illustrating cytoskeleton of control and Elafin silencing cells. Scale bar, 20 μm. **f** Representative immunofluorescence images of EMT markers expression in Elafin silencing cells. Scale bar, 50 μm. **g** Expression of EMT markers mediated by Elafin silencing are shown by western boltting. **h** Down-regulation of Elafin significantly suppressed lung metastasis in nude mice model established by injection of indicated cells through the tail vein. Representative HE staining images are shown. Scale bar, 100 μm. **i** Lung metastasis nodules from the nude mice model are analyzed. *** *P* < 0.001

We next evaluated the functions of Elafin in HCC cells. We first examined the expression of Elafin in HCC cell lines and found that Elafin was predominantly present in the conditioned medium, but was barely detected in the whole-cell lysates (Fig. S1B and C). Then, we constructed stable Elafin knockdown PLC-8024 and Huh7 cells using two shRNAs (Fig. 2a).

To determine whether Elafin regulates the growth of HCC cells, cell proliferation and colony formation assays were performed, and no significant changes were observed between Elafin knockdown and control cells (Fig. S2). Then, we performed migration and invasion assays and scratch wound healing assays and found that silencing of Elafin significantly suppressed the migration, invasion, and wound healing ability of HCC cells (Fig. 2b, c, and d). Accumulating evidence has emphasized that EMT plays a vital role in cancer metastasis [27, 28]. Therefore, we wondered whether Elafin promoted the migration and invasion of HCC cells by triggering EMT. Interestingly, we found that Elafin knockdown cells exhibited a cobblestone or shrunken shape based on the staining of F-actin fibres (Fig. 2e), which suggested that Elafin might influence the transition of HCC cells from the epithelial phenotype to the mesenchymal phenotype. Furthermore, IF staining of EMT markers showed that the expression of the epithelial marker E-cadherin was increased and the expression of the mesenchymal marker vimentin was decreased in Elafin knockdown cells compared to control cells (Fig. 2f), and western blotting assays were performed to verify these findings (Fig. 2g). Consistent with the above findings, smaller and fewer metastatic lung nodules were observed in the Elafin knockdown group than that in the control group in our tail vein lung metastasis mouse model (Fig. 2h and i). These results indicated that Elafin knockdown inhibited the EMT and metastasis of HCC cells.

### Secreted Elafin induces the EMT and metastasis of HCC cells in vitro and in vivo

**Fig. 3:**
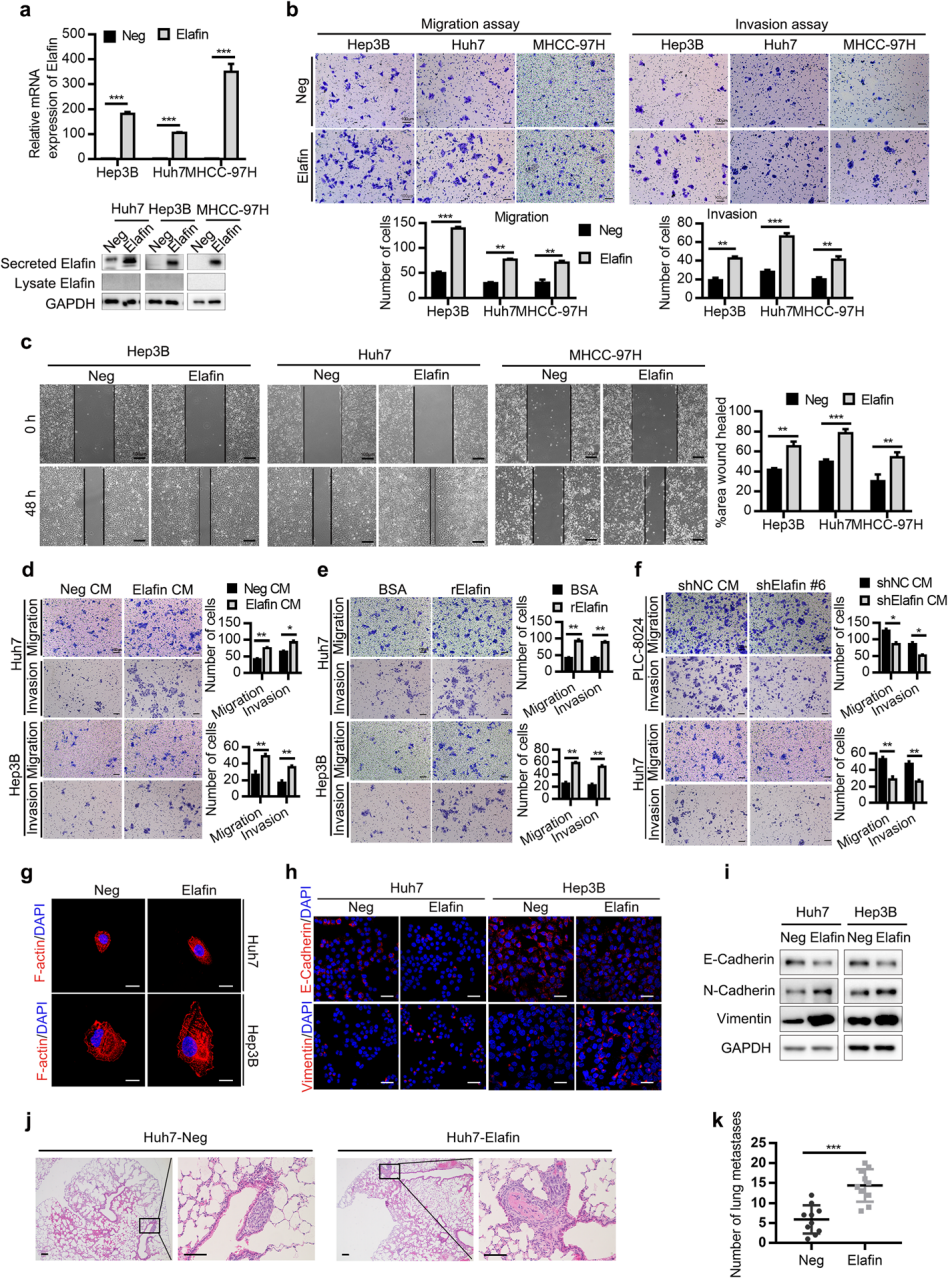
Overexpression of Elafin promotes EMT and metastasis of HCC cells in vitro and in vivo. **a** The efficiency of Elafin overexpression were measured by real-time PCR (top) and Western blotting (bottom) assays. *** *P* < 0.001. **b** Overexpression of Elafin enhanced migration and invasion of MHCC-97H, Huh7 and Hep3B cells. Scale bar, 100 μm. Statistical results are presented as mean ± SD (from triplicates), and significance is determined by Student *t* test (*** *P* < 0.001). **c** Overexpression of Elafin enhanced the scratch wound-healing ability of MHCC-97H, Hep3B and Huh7 cells. Scale bar, 100 μm. Statistical results at 48 h are presented as mean ± SD (from triplicates) (** *P* < 0.01; *** *P* < 0.001). **d** and **e** Concentrated Elafin-overexpression condition medium (Elafin CM) and commercial recombinant Elafin (rElafin, 10 μg/ml) promoted migration and invasion of wild-type HCC cells. Scale bar, 100 μm. Statistical results are presented as mean ± SD (from triplicates) (* *P* < 0.05; ** *P* < 0.01;*** *P* < 0.001). **f** The effects of concentrated Elafin-knockdown conditioned medium on HCC cells. Scale bar, 100 μm. Statistical results are presented as mean ± SD (from triplicates) (* *P* < 0.05; ** *P* < 0.01). **g** Representative immunofluorescence images illustrating cytoskeleton of control and Elafin overexpressing cells. Scale bar, 20 μm. **h** Representative immunofluorescence images of EMT markers expression in Elafin overexpressing cells. Scale bar, 50 μm. **i** Expression of EMT markers mediated by Elafin overexpression are shown by western boltting. **j** Overexpression of Elafin significantly enhanced lung metastasis in nude mice model established by injection of indicated cells through the tail vein. Representative HE staining images are shown. Scale bar, 100 μm. **k** Lung metastasis nodules from the nude mice model are analyzed. *** *P* < 0.001

To further verify the function of Elafin in HCC cells, we constructed stable Elafin overexpressing MHCC-97H, Hep3B and Huh7 cells (Fig. 3a). In accordance with the findings in the Elafin knockdown cells, overexpression of Elafin had no effects on HCC cell proliferation (Fig. S3) but enhanced the metastasis of HCC cells (Fig. 3b and c). In addition, after stimulation with conditioned medium collected from stable Elafin overexpressing HCC cells or commercial recombinant Elafin, HCC cells exhibited increased migration and invasion abilities (Fig. 3d and e), while after stimulation with the conditioned medium collected from the Elafin knockdown groups, the HCC cells exhibited lower migration and invasion abilities compared with the negative control conditioned medium (Fig. 3f). These results suggested that Elafin could promote HCC metastasis in a paracrine or autocrine manner. IF staining showed that Elafin overexpressing cells exhibited a more spindle or elongated shape than that of negative control cells (Fig. 3g). Furthermore, E-cadherin was downregulated and vimentin was upregulated in Elafin overexpressing cells (Fig. 3h and i). In the tail vein lung metastasis mouse model, the number of metastatic lung nodules was remarkably increased in the Elafin overexpressing group compared with the negative control group (Fig. 3j and k). These results indicated that Elafin induced the EMT and metastasis of HCC cells.

### Elafin interacts with EGFR and activates EGFR/AKT signalling

**Fig. 4:**
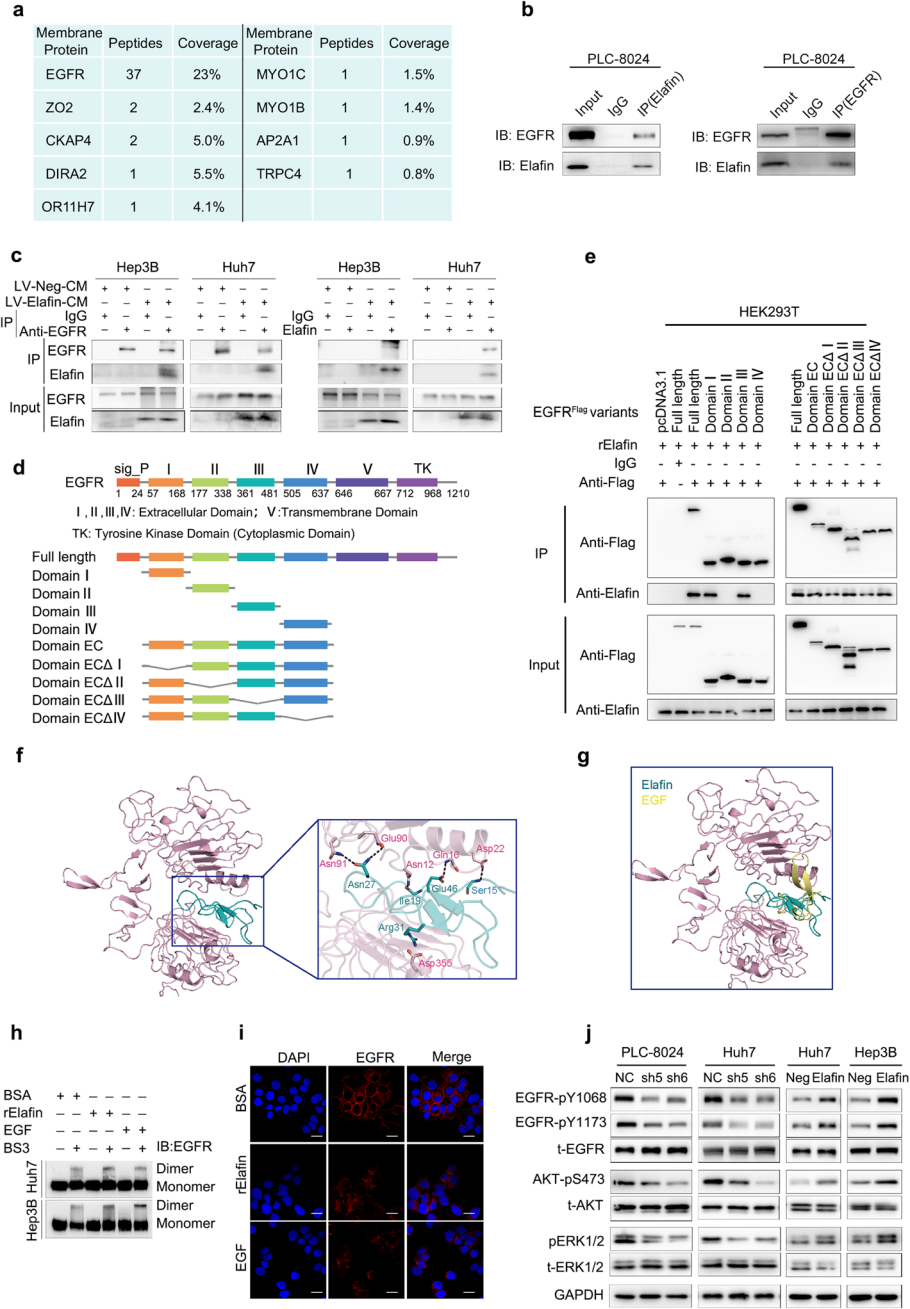
Elafin interacts with EGFR, promotes EGFR dimerization and internalization, and activates EGFR/AKT pathway. **a** Mass spectrometry analysis of Elafin-associated proteins in PLC-8024 cells. The list of peptide fragments (95% CI) and peptide coverage (95% CI) of membrane proteins is presented. **b** Endogenous Elafin interacted with EGFR in PLC-8024 cells. The whole-cell lysate and indicated concentrated condition medium were mixed and then immunoprecipitated. After separation on SDS gels, western blotting was conducted with the indicated antibodies. **c** Co-immunoprecipitated was performed to detect the interaction between Elafin and endogenous EGFR in Elafin overexpressing HCC cells. The whole-cell lysate was mixed up with concentrated vector or Elafin-overexpression condition medium and then were immunoprecipitated. **d** Schematic diagram of wild-type EGFR and its truncated mutants was shown. **e** Elafin could bind to the EGFR extracellular domain via subdomain I and III. HEK293T cells were transfected with indicated EGFR constructs and then lysed at 48-h. After incubation with the rElafin, the lysates were subjected to co-IP assays. **f** The putative binding mode of Elafin and EGFR. EGFR and Elafin were shown as light pink cartoon and teal cartoon, respectively. **g** Elafin bound to EGFR at the same binding site for EGF. EGFR, Elafin and EGF were shown as light pink cartoon, teal cartoon and yellow cartoon, respectively. **h** Induction of EGFR dimerization of Elafin. After starving and treatment with BSA as control, rElafin (10 μg/ml) or EGF (100 ng/ml), the cells were crosslinked with 3 mM BS^3^ and were subjected to western blotting assays. **i** rElafin promoted EGFR internalization. Huh7 cells were starved for 24 h and then treated with BSA, rElafin and EGF for 30 min, then immunofluorescence staining was performed. Scale bar, 20 μm. **j** Key members of EGFR downstream signaling expressions were detected by western blotting in Elafin knockdown and overexpressing HCC cells

According to the above results, we speculated that secreted Elafin might exert its functions by binding to receptors on the cell surface. Therefore, we performed immunoprecipitation and mass spectrometry to identify the interacting proteins of Elafin using the PLC-8024-cell-mixture (PLC-8024 cell extracts mixed with PLC-8024 concentrated conditioned medium). Numerous Elafin-interacting membrane proteins were identified (Table S6), among which EGFR showed a high probability of interacting with Elafin (Fig. 4a). Next, co-immunoprecipitation experiments conducted in the PLC-8024-cell-mixture showed that Elafin could physically interact with EGFR (Fig. 4b). Importantly, similar results were also found in Elafin overexpressing HCC cells, which verified that Elafin and EGFR could interact with each other in HCC cells (Fig. 4c).

To map the binding domain of EGFR responsible for Elafin interaction, a series of constructs encoding EGFR individual subdomain and EGFR mutants with subdomain deletions were generated (Fig. 4d). Co-IP and western blotting assays showed that Elafin could bind to the EGFR extracellular domain via subdomain I and III (Fig. 4e). Furthermore, we also performed molecular docking assays to identify the binding mode mediating the interaction between Elafin and EGFR. Interestingly, Elafin was situated in the cavity formed by domain I and domain III of the EGFR ectodomain (Fig. 4f), which resembled the binding mode of EGF and EGFR (Fig. 4g). In detail, a salt bridge formed between the Arg31 residue of Elafin and the Asp355 residue of domain III of EGFR as well as the hydrogen-bond interactions established within the Asn27, Ile19, Glu46, Ser15, and Arg31 residues of Elafin and the Asn91, Glu90, Asn12, Gln16, and Asn22 residues of domain I of EGFR mediated the binding of Elafin and EGFR (Fig. 4f). These results indicated that the domain I and domain III were crucial for the interaction between EGFR and Elafin.

Given that Elafin interacts with EGFR, we then sought to investigate whether Elafin could activate EGFR signalling. First, we performed BS^3^ cross-linking assays and found that commercial recombinant Elafin (rElafin) and EGF could similarly stimulate the formation of EGFR dimers (Fig. 4h). Meanwhile, IF staining showed that the EGFR levels on the cell surface decreased after rElafin and EGF stimulation (Fig. 4i). These findings indicated that Elafin could induce EGFR dimerization and internalization in a manner similar to that of EGF, the classic ligand of EGFR. Similar to EGF, we observed that rElafin could activate EGFR by elevating EGFR phosphorylation at the Y1068 and Y1173 sites and also induce a more spindle or elongated cell shape (Fig. S4A and B). Furthermore, the levels of EGFR phosphorylation at Y1068 and Y1173 and AKT and ERK1/2 phosphorylation were increased in Elafin overexpressing cells, whereas Elafin knockdown decreased their phosphorylation (Fig. 4j). We also examined other recognized downstream markers of EGFR, such as JNK, MAPK, GSK3β, and STAT3, however, no significant changes were observed in either Elafin knockdown or overexpressing HCC cells (Fig. S5). In summary, these results revealed that Elafin could bind to EGFR and activate EGFR signalling.

### Elafin facilitates EMT and HCC metastasis via EGFR/AKT signalling

**Fig. 5:**
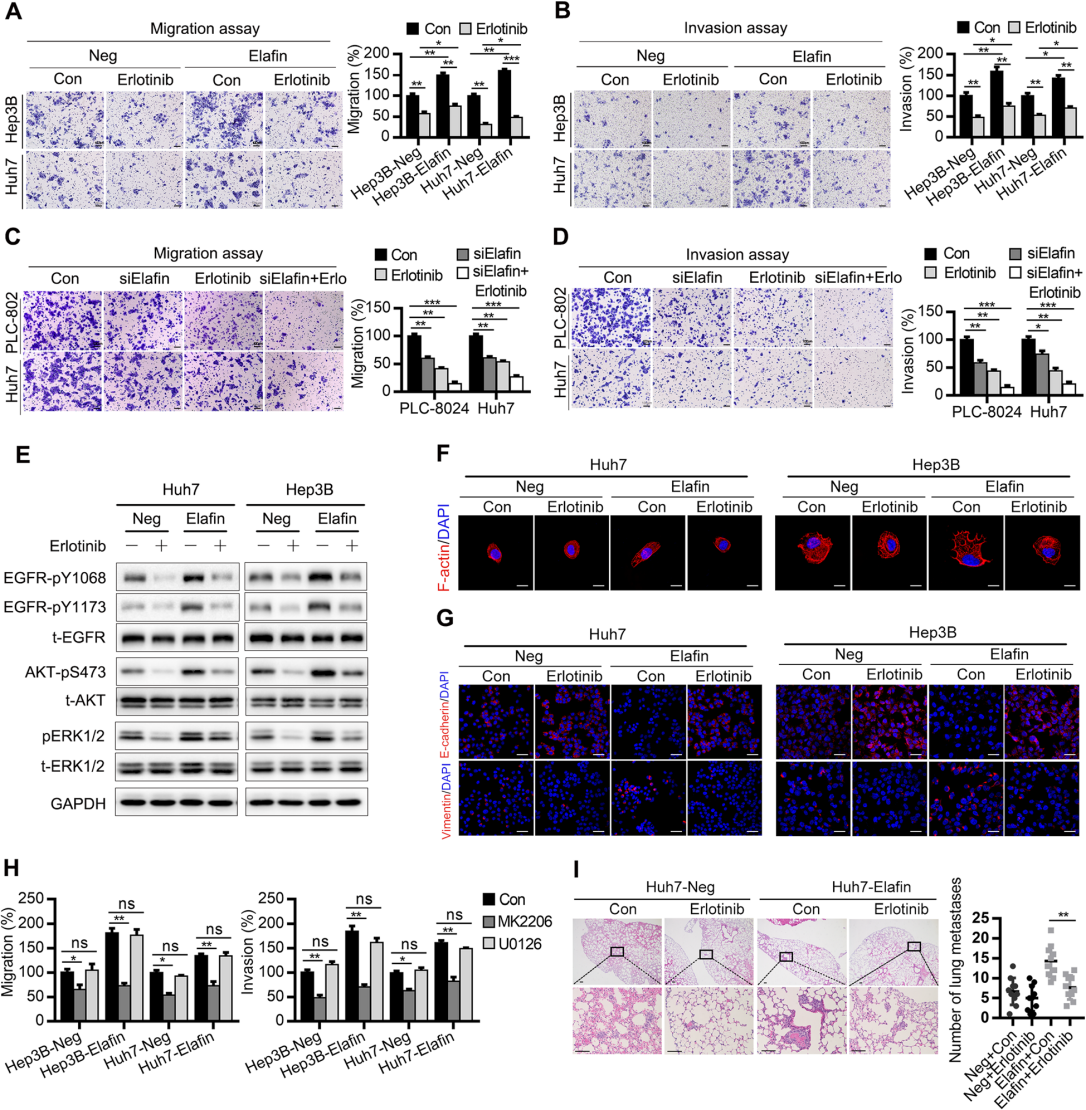
Elafin induces EMT and metastasis though EGFR/AKT signaling independently and attenuated the effect of erlotinib in HCC. **a** and **b** Inhibitor of EGFR impaired migration (**a**) and invasion (**b**) of Elafin-overexpressing HCC cells. Cells were treated with Erlotinib (3 μM) for 24 h and then were subjected to trans-well assays. Scale bar, 100 μm. Statistical results are presented as mean ± SD (from triplicates), and significance is determined by Student *t* test (* *P* < 0.05; ** *P* < 0.01; *** *P* < 0.001). **c** and **d** Down-regulated of Elafin enhanced the effect of Erlotinib on suppressing migration (**c**) and invasion (**d**) in HCC cells. After treated with siRNA of Elafin, erlotinib (3 μM), and both of them, indicated cells were conducted trans-well assays. Statistical results are presented as mean ± SD (* *P* < 0.05; ** *P* < 0.01; *** *P* < 0.001). **e** Erlotinib inhibited phosphorylation of EGFR and downstream signaling. Indicated cells were treated with erlotinib (3 μM) for 24 h and then performed western blotting. **f** Representative immunofluorescence images illustrating cytoskeleton of control and Elafin overexpressing cells after treated with Erlotinib (3 μM) for 24 h. Scale bar, 20 μm. **g** Representative immunofluorescence images of EMT markers expression in Elafin overexpressing cells after treated with Erlotinib (3 μM) for 24 h. Scale bar, 50 μm. **h** The effects of inhibitors of AKT and ERK on the migration and invasion of Elafin-overexpressing HCC cells. Cells were treated with MK2206 (1 μM) and U0126 (15 μM) for 12 h and then were subjected to transwell assays. Statistical results are presented as mean ± SD (ns, no significance; * *P* < 0.05; ** *P* < 0.01; *** *P* < 0.001). **i** Erlotinib impaired lung metastases of Elafin-overexpressing HCC cells in vivo. Representative HE staining images are shown. Scale bar, 100 μm. Quantification of lung metastasis nodules is analyzed. ** *P* < 0.01

To further evaluate whether EGFR, AKT, or ERK1/2 signalling is essential for Elafin-induced HCC metastasis, specific inhibitors of EGFR, AKT, or MEK/ERK were applied for the rescue experiments (Fig. S6A and B). We found that erlotinib dramatically attenuated the migration, invasion and wound healing abilities of Elafin overexpressing cells (Fig. 5a and b; Fig. S7A and B). Moreover, combining erlotinib treatment with Elafin knockdown using specific Elafin siRNA (Fig. S8) showed synergetic inhibitory effects on migration and invasion in HCC cells (Fig. 5c and d). Mechanistically, erlotinib treatment could decrease the phosphorylation of EGFR and downstream AKT and ERK1/2 (Fig. 5e). In addition, erlotinib suppressed the mesenchymal phenotype induced by ectopic Elafin expression (Fig. 5f and g). Importantly, an inhibitor of AKT, MK2206, also attenuated the migration and invasion abilities of Elafin overexpressing cells, while an inhibitor of MEK/ERK1/2, U0126, did not (Fig. 5h; Fig. S6C). Consistently, in our lung metastasis mouse model, the number of metastatic nodules markedly reduced after erlotinib treatment (Fig. 5i). These results indicated that Elafin promoted HCC cell metastasis via the EGFR/AKT pathway independently.

### Elafin attenuates the suppressive effects of erlotinib on HCC metastasis

The above findings showed that erlotinib impaired Elafin-induced HCC metastasis. However, compared with the negative vector cells when treated with erlotinib, Elafin overexpressing cells still exhibited greater migration and invasion abilities and were more likely to exhibit a mesenchymal phenotype (Fig. 5a, b, and f). Moreover, combining erlotinib treatment with Elafin knockdown showed synergetic inhibition of migration and invasion in HCC cells (Fig. 5c and d). Consistent with these findings, after treatment with erlotinib, the EGFR/AKT signalling in Elafin overexpressing cells remained more activated than that in negative vector cells (Fig. 5e). More importantly, in the oral erlotinib mouse groups, the number of lung metastasis nodules derived from Elafin overexpressing cells was higher than that derived from negative vector cells (Fig. 5i). These results indicated that Elafin attenuated the effects of erlotinib and that silencing of Elafin enhanced the suppressive effects of erlotinib on HCC metastasis.

### PI3 (Elafin) is transcriptionally regulated by Sp1

**Fig. 6:**
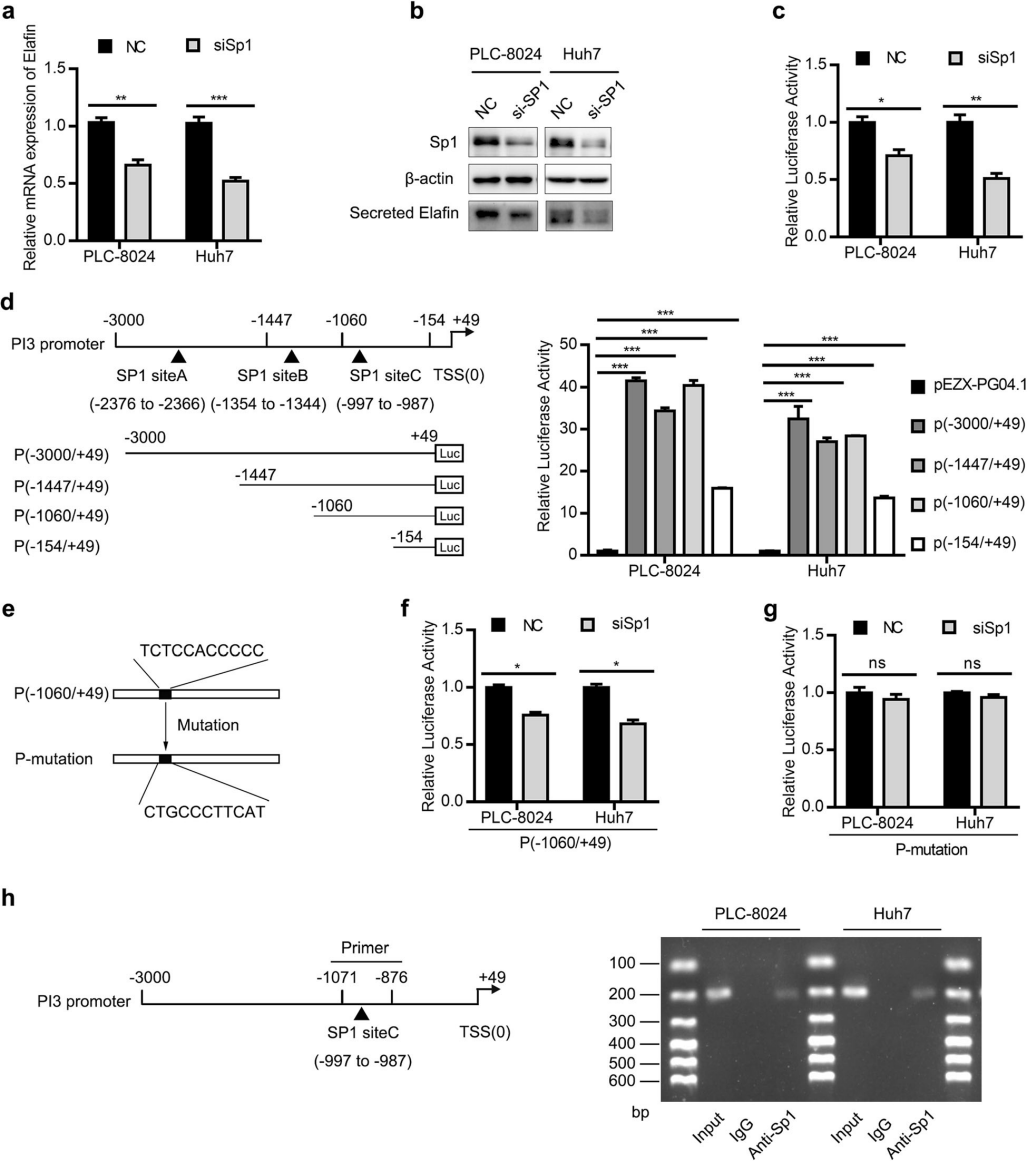
Sp1 regulates Elafin expression by activating Elafin promoter. **a** and **b** Sp1 decreased Elafin expression in HCC cells. Real-time PCR assay (**a**) and western blotting assay (**b**) were performed to detect Sp1 and Elafin expression after transfected with NC or siRNA of Sp1. ** *P* < 0.01, *** *P* < 0.001. **c** Knockdown of Sp1 reduced the Elafin promoter activity. HCC cells were reversely transfected with NC or siSp1 for 24 h, followed by transfection with full-length Elafin promoter (P (− 3000/+ 49) for another 48 h, then were subjected to luciferase activity analysis. * *P* < 0.05, ** *P* < 0.01. **d** Schematic view of the luciferase reporter constructs containing various length of the 5′-flanking regions of the Elafin encoding gene, PI3. Detailed characterization of the PI3 promoter by 5′-deletion and site-specific deletion analyses were performed. HCC cells were transfected with the vector (PEZX-PG04.1) and indicated luciferase reporter constructs for 48 h, then was subjected to luciferase activity analysis. *** *P* < 0.001. **e** The sequence of siteC in P (− 1060/+ 49) and the corresponding sequence of mutation in P-mutation are presented. **f** Knockdown of Sp1 decreases the luciferase activity of P (− 1060/+ 49). HCC cells were reversely transfected with NC or siSp1 for 24 h, followed by transfection with (P (− 1060/+ 49) for another 48 h, then were subjected to luciferase activity analysis. * *P* < 0.05. **g** Knockdown of Sp1 do not change the luciferase activity of P-mutation. HCC cells were reversely transfected with NC or siSp1 for 24 h, followed by transfection with P-mutation for another 48 h, then were subjected to luciferase activity analysis. **h** Sp1 directly bound to the PI3 promoter. Schematic representation of PI3 promoter regions and indicated primers for CHIP assay (left) were conducted and CHIP samples were analyzed by semi-quantitive PCR (right)

Previous studies have revealed that the differential expression of Elafin in human normal epithelial cells and carcinomas is regulated at the transcriptional level [29]. Thus, we analysed the upstream region of PI3 (+ 49 to − 3000 kb) in JASPAR (http://jaspar.genereg.net/). Using 10 scores as a cutoff point and 80% as the relative profile score threshold, three binding sites of Sp1 were predicted in the putative promoter region of PI3 (Table S8). Importantly, the mRNA and protein expression levels of Elafin were significantly downregulated after specifically silencing Sp1 with siRNA (Fig. 6a and b). Promoter luciferase assays showed that the transcriptional activity of the full-length PI3 promoter was positively changed when Sp1 was silenced (Fig. 6c). To further identify the functional Sp1 binding regions in the PI3 promoter, various lengths of the PI3 5′-flanking region were constructed, and the subsequent luciferase reporter assays revealed markedly decreased luciferase activity in the P (− 154/+ 49) construct compared with the full-length and the other two constructs, P (− 1447/+ 49) and P (− 1060/+ 49) (Fig. 6d). These findings suggested that nucleotides (nts) -1060/− 154 of the PI3 promoter region, where one Sp1 predicting binding site (Sp1 site C) existed, is vital for Sp1 to elicit transcriptional responses. Therefore, we generated a P-mutation construct, which contained a mutated site C, for the next experiments (Fig. 6e). Moreover, Sp1 silencing significantly reduced the luciferase activity of the P (− 1060/+ 49) construct, while the luciferase activity of the P-mutation construct with mutated Sp1 site C remained similar to that of the control group (Fig. 6f and g). Then, we performed a ChIP assay to examine the physical interaction of Sp1 with the PI3 promoter, and the semiquantitative PCR analyses indicated that Sp1 physically binds to the PI3 promoter (Fig. 6h). These results showed that Sp1 transcriptionally regulated PI3 expression in HCC cells.

### Elafin-mediated EGFR/AKT activation predicts outcomes in HCC patients

**Fig. 7:**
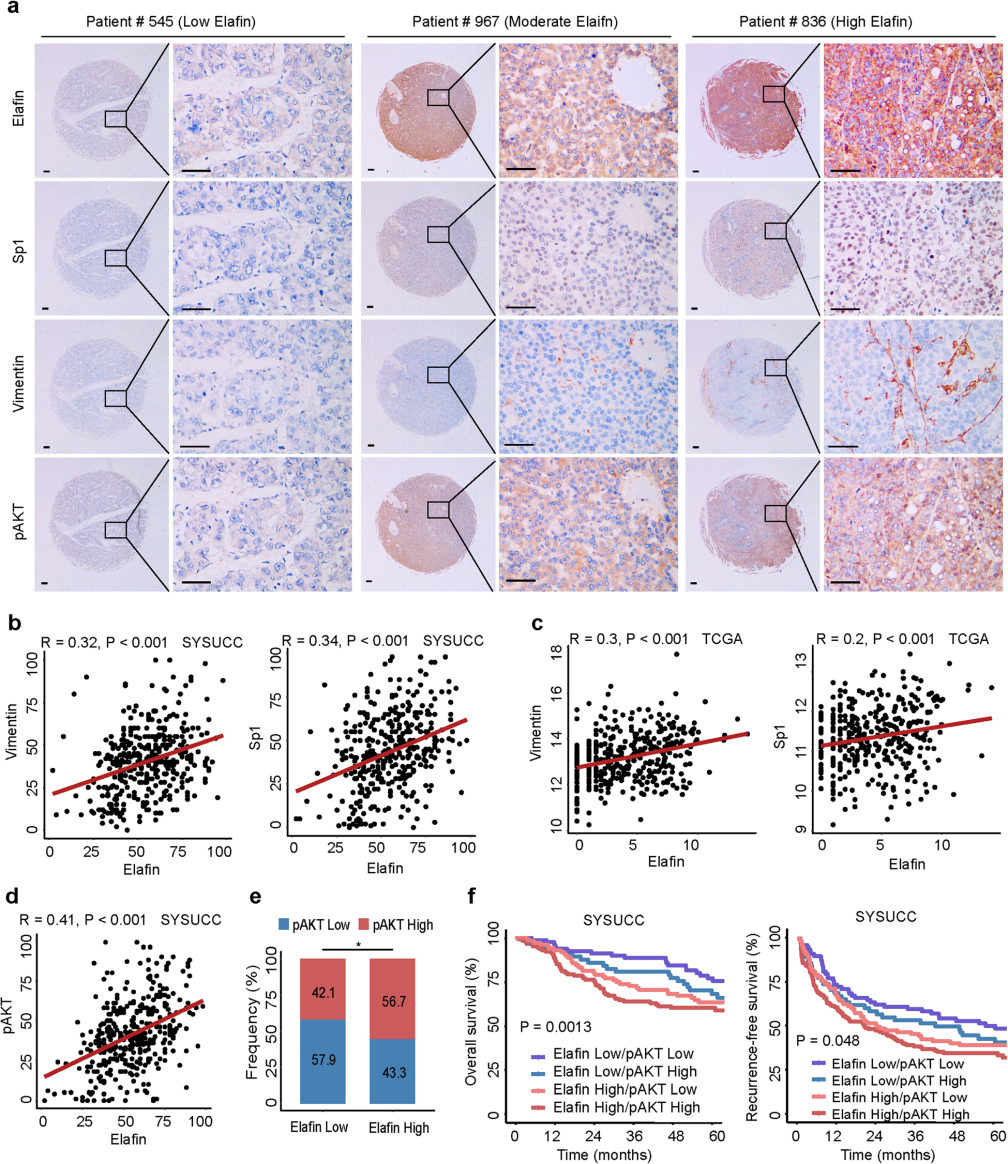
Association between Elafin, activated AKT, Vimentin, and Sp1 in human HCC tissues. **a** Representative immunohistochemical staining images of pAKT, Vimentin, and Sp1 in different levels of Elafin expression HCC tissues. Scale bar, 100 μm. **b** Positive correlations between Elafin and Vimentin or Sp1 protein levels in HCC tissues based on IHC scores (SYSUCC cohort, *n* = 378). R and *P* value were measured using Pearson’s correlation test. **c** Positive correlations between Elafin and Vimentin or Sp1 mRNA expressions based on TCGA dataset (*n* = 371). R and P value were measured using Pearson’s correlation test. **d** and **e** Pearson’s correlation (**d**) and stacked bar graph (**e**) showed that Elafin expression was positively associated with pAKT expression based on IHC scores. * *P* < 0.05. **f** The combination of Elafin and pAKT increased the probability of a poor prognosis. OS and RFS curves for patients with combination Elafin with pAKT expression in SYSUCC cohort (*n* = 378) are depicted

Owing to the results illustrated in the HCC cells and the mouse model, we then investigated the association between the expression of Elafin and those of pAKT, vimentin, and Sp1 in HCC specimens. Representative images of IHC staining showed that HCC patients with low expression of Elafin usually exhibited relatively low expression of pAKT, vimentin, and Sp1. In contrast, HCC patients with high expression of Elafin frequently manifested relatively high expression of pAKT, vimentin, and Sp1 (Fig. 7a). Based on IHC scores, a positive correlation between the expression of Elafin and vimentin (*r* = 0.32; *P* < 0.001) or Sp1 (*r* = 0.34; *P* < 0.001) was identified (Fig. 7b). Consistently, analysis of the TCGA database verified the correlation between Elafin and vimentin (*r* = 0.3; *P* < 0.001) and the correlation between Elafin and Sp1 (*r* = 0.2; *P* < 0.001) (Fig. 7c).

We then analysed the expression of Elafin and pAKT based on IHC scores, and a strong positive correlation (*r* = 0.41; *P* < 0.001; Fig. 7d and e) inspired us to establish a prognostic risk stratification. The Kaplan-Meier curves showed that HCC patients with increased expression of both Elafin and pAKT had the shortest OS and RFS, while HCC patients with low expression of both Elafin and pAKT exhibited the best outcomes (Fig. 7f). In summary, these clinical results revealed that the expression of Elafin was positively correlated with the expression of pAKT, vimentin, and Sp1 in HCC and that combined assessment of Elafin and pAKT expression could increase the efficiency of HCC prognosis prediction.

## Discussion

**Fig. 8:**
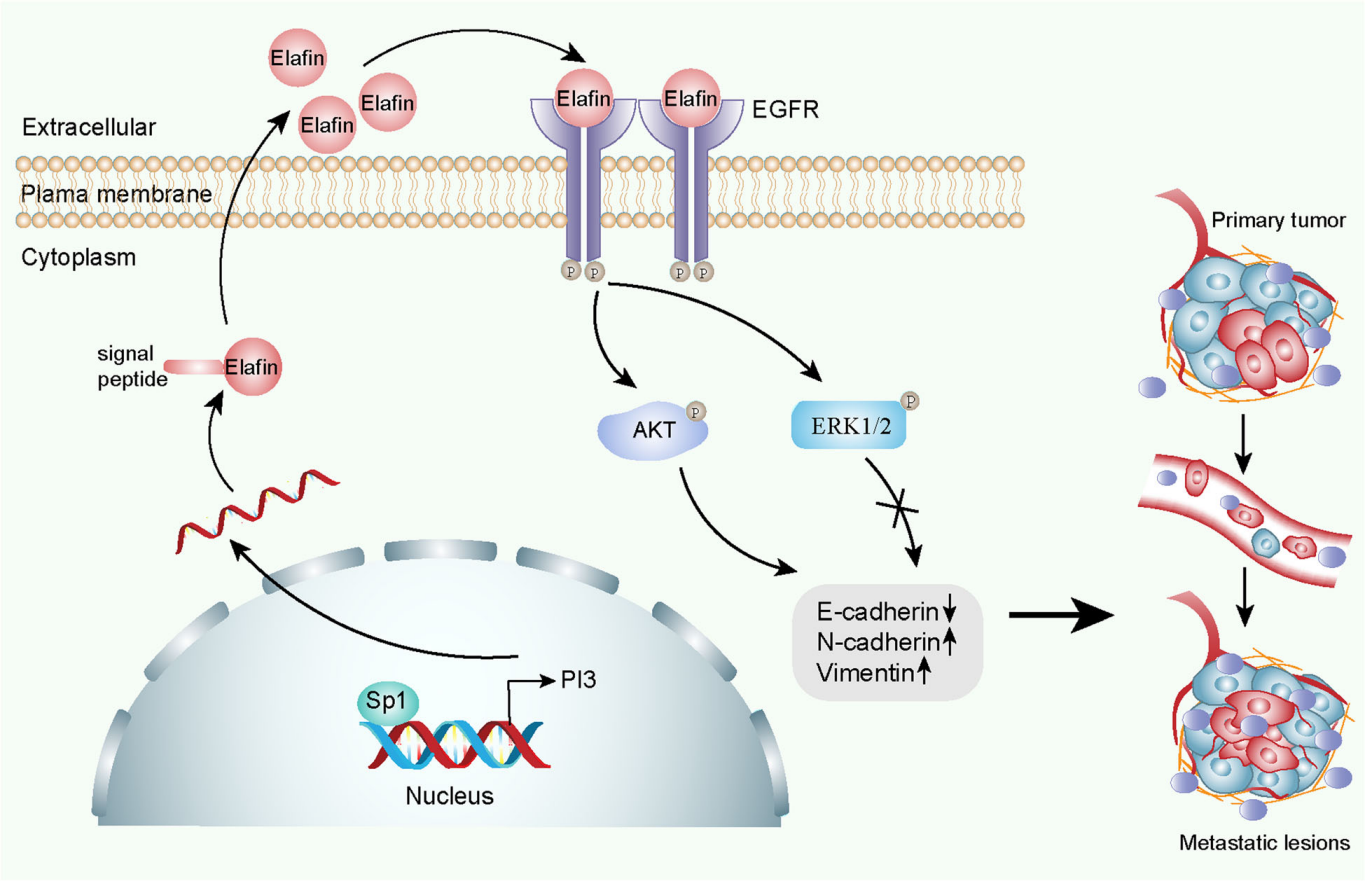
Schematic diagram of the underlying mechanisms up-regulation of Elafin by Sp1 enhances HCC metastasis and promotes Erlotinib resistance via EGFR/AKT signalling

In the present study, we found that Elafin was commonly upregulated in HCC and that increased Elafin expression indicated a poor prognosis in HCC patients. Moreover, Elafin contributed to the transition to a mesenchymal phenotype and enhanced the metastasis of HCC cells. Mechanistically, Elafin interacted with EGFR and activated EGFR/AKT signalling (Fig. 8). Importantly, Elafin attenuated the effects of erlotinib on HCC treatment by regulating EGFR signalling. To our knowledge, our study is the first to report that Elafin may serve as an effective molecular target for blocking HCC metastasis.

Elafin plays important roles in homoeostasis and inflammation [12]. Dysfunction of Elafin is involved in the progression of many diseases, such as innate and adaptive immunity diseases [30, 31], inflammatory lung diseases [32], and cardiovascular injury [33]. Recently, growing evidence has revealed the relationship between Elafin and malignancies, indicating that Elafin could be a potential molecular marker for several cancers [14]. In agreement with the results in breast cancer and ovarian cancer [15, 34], our results showed that Elafin was elevated in HCC tissues compared to normal tissues and that increased Elafin expression predicted a poor prognosis in HCC patients. In addition, high Elafin expression was significantly associated with aggressive tumour features. These results encouraged us to further investigate the exact role of Elafin in HCC.

According to previous studies, Elafin plays dual roles in cancer. Several studies stated that Elafin inhibited cell proliferation by mediating retinoblastoma-dependent growth arrest or caspase-3-or p53-dependent apoptosis [18–20], whereas several studies reported that Elafin promoted cell proliferation through activation of MAPK or NF-κB pathway [15, 34]. However, in this study, Elafin did not affect cell proliferation but facilitated EMT and metastasis of HCC cells. These results further verified that Elafin might play different roles in various malignancies. Although the role of EMT in cancer metastasis is controversial [35, 36], numerous studies have emphasized the crucial role of EMT in the early-stage dissemination of cancer cells and in metastasis and chemoresistance [37, 38]. In the present study, Elafin induced HCC metastasis by triggering the transition of cells from the epithelial phenotype to the mesenchymal phenotype, which further emphasized the critical role of EMT in HCC. In summary, this study is the first to illustrate that Elafin induces EMT in HCC and promotes HCC metastasis.

As a secreted protein, Elafin might exhibit its functions by binding to receptors on the cell surface. Hence, Co-IP and mass spectrometry assays were performed, and EGFR was identified as a candidate receptor. Furthermore, we verified that Elafin could interact with EGFR and activate EGFR phosphorylation. EGFR, a kind of growth factor receptor with tyrosine kinase activity (RTK), has been demonstrated to play a crucial role in the tumorigenesis and progression of many solid tumours [39]. In terms of HCC, approximately 40–70% of patients exhibit EGFR overexpression and EGFR gene copy number amplification [40]. Thus, we further investigated the role of Elafin and EGFR in HCC progression and found that Elafin could activate EGFR and the downstream AKT and ERK1/2 pathways. Recently, one study stated that Elafin promoted the proliferation of cancer cells through the ERK1/2 pathway in ovarian and breast cancer [15], which partly supports our results. Furthermore, rescue experiments showed that erlotinib and MK2206, but not U0126, could abrogated the Elafin induced metastasis of HCC cells. These results revealed that Elafin promoted HCC metastasis via EGFR/AKT pathway independently. To our knowledge, we are the first to identify Elafin as an oncogenic regulator of EGFR/AKT pathway activation by binding to EGFR in HCC. These findings urged us to investigate the role of Elafin in erlotinib therapy for HCC.

As the only phase III clinical trial, the SEARCH trial, has failed to achieve better outcomes in advanced HCC [9], and accumulating studies have focused on the exact regulatory mechanisms of EGFR in HCC. Several studies have stated that various molecules, such as choline kinase alpha (CHKA), reticulocalbin-2 (RCN2), MUC15, and versicanV1, could promote HCC proliferation or metastasis and induce EGFR antagonist resistance by interacting with EGFR and activating the EGFR signalling [41–44]. Consistent with these studies, we found that erlotinib could partly inhibit Elafin induced HCC metastasis and EGFR/AKT signalling activation, and the combination of erlotinib treatment and Elafin knockdown showed a synergetic suppressive effect on HCC metastasis. These findings indicated that Elafin contributed to impairing the effects of erlotinib and that silencing of Elafin synergistically strengthened the inhibitory effects of erlotinib on HCC metastasis. Thus, in the future, drugs or molecules targeting Elafin could be researched and explored, and combination therapy consisting of anti-Elafin and anti-EGFR compounds could be applied in EGFR-dependent HCC therapy.

Elafin has been reported to be regulated at the transcriptional level by numerous transcription factors, including NF-κB and AP-1 [29, 34, 45]. Specificity protein 1 (Sp1) is one of the most well-characterized transcriptional activators and regulates a variety of genes that are involved in cell growth, differentiation, apoptosis, and carcinogenesis [46]. Although Sp1 was previously predicted to bind to the promoter of PI3 (Elafin) [29, 34], no evidence has verified the relationship between Sp1 and Elafin. To fill this gap, we investigated this and found that Sp1 could bind to the promoter of PI3 and regulate the expression of PI3 in HCC. In addition, several studies have shown that Sp1 induces cancer metastasis by regulating the expression of CD147 [47] or affecting EMT by upregulation of SNAIL expression [48], which also supports our findings. This is the first study to verify that Sp1 could transcriptionally regulate the expression of Elafin and to explain why Elafin is upregulated in HCC tissues.

## Conclusions

In summary, we identified Elafin as a key promoter of HCC metastasis. By binding to EGFR, Elafin triggered HCC metastasis via activating EGFR/AKT signalling. More importantly, silencing Elafin synergistically strengthened the anti-metastatic effects of erlotinib. Thus, our findings provide a valuable prognostic biomarker and a novel therapeutic target for HCC, and thus, combining inhibitors or neutralizing antibodies targeting Elafin with anti-EGFR drugs may be a promising approach for EGFR-positive HCC.

## Supplementary Information

**Additional file 1.** Supplementary Materials and Methods.

## Data Availability

All data generated or analyzed during this study are included either in this article or in the supplementary information files. The bioinformatic data was deposited in the Cancer Genome Atlas (TCGA)-Liver Hepatocellular Carcinoma (LIHC) and Gene Expression Omnibus (GEO) database (https://www.ncbi.nlm.nih.gov/geo/) with an accession number: GSE 14520.

## Abbreviations

HCC: Hepatocellular carcinoma
EGFR: Epidermal growth factor receptor
EMT: Epithelial-to-mesenchymal transition
PI3: Peptidase inhibitor 3
SCAND3: SCAN domain containing 3
SGIP1: Src homology 3-domain growth factor receptor-bound 2-like interacting protein 1
SKALP: Skin-derived antileukoproteinase
WAP: Whey acidic protein family
SYSUCC: Sun Yat-sen Cancer Center
DMEM: Dulbecco’s Modified Eagle medium
FBS: Fetal bovine serum
IHC: Immunohistochemical staining
IF: Immunofluorescence staining
Co-IP: Co-immunoprecipitation assay
Gluc: Gaussia luciferase
SEAP: Secreted alkaline phosphatase
TCGA: The Cancer Genome Atlas
GEO: Gene Expression Omnibus
OS: Overall survival
RFS: Recurrence-free survival
AFP: Alpha-fetoprotein
HR: Hazard ratio
CI: Confidence interval
EGF: Epidermal growth factor
CHIP: Chromatin immunoprecipitation assay
Rb: Retinoblastoma
NF-κB: nuclear factor κB
RTKs: Tyrosine kinase activity
CHKA: Choline kinase alpha
RCN2: Reticulocalbin-2
Sp1: Specificity protein 1
