# Correction to: Elafin promotes tumour metastasis and attenuates the anti-metastatic effects of erlotinib via binding to EGFR in hepatocellular carcinoma

**DOI:** 10.1186/s13046-022-02313-5

**Published:** 2022-03-18

**Authors:** Chenwei Wang, Yadi Liao, Wei He, Hong Zhang, Dinglan Zuo, Wenwu Liu, Zhiwen Yang, Jiliang Qiu, Yichuan Yuan, Kai Li, Yuanping Zhang, Yongjin Wang, Yunxing Shi, Yuxiong Qiu, Song Gao, Yunfei Yuan, Binkui Li

**Affiliations:** 1 State Key Laboratory of Oncology in South China, Collaborative Innovation Center for Cancer Medicine, Sun Yat-sen University Cancer Center, Guangzhou, 510060 People’s Republic of China; 2 Department of Liver Surgery, Sun Yat-sen University Cancer Center, 651 Dongfeng Road East, Guangzhou, 510060 People’s Republic of China

**Correction to: J Exp Clin Cancer Res 40, 113 (2021)**

**https://doi.org/10.1186/s13046-021-01904-y**

Following publication of the original article [1], the authors identified minor errors in Figs. 2, 3 and 5, specifically:

- Fig. 2d: image for PLC-8024 shElafin #6 at 0 h (1^st^ row, 3^rd^ column); correct image now used
- Fig. 3b: image for the invasion assay of Huh7 Neg and Elafin (1^st^ and 2^nd^ row, 5^th^ column); correct images now used
- Fig. 3d: image for the invasion assay of Huh7 Neg CM (2^nd^ row, 1^st^ column); correct image now used
- Fig. 5g: images for the E-cadherin and Vimentin of Huh7-Elafin-Con (1^st^ and 2^nd^ row, 3^rd^ column), and the E-cadherin of Hep3B-Neg-Con (1^st^ row, 5^th^ column); correct images now used

In addition, the authors revised the Supplemental Figures file during manuscript revision, but the original file was published alongside the article. The revised version has now been used, with the following changes from the original:

- Fig. S1c, additional western blots included to show Elafin protein levels in whole cell lysate (top) and condition medium (bottom)
- Fig. S6c, correct images used for invasion assay of Hep3B-Neg-U0126 (1^st^ row, 3^rd^ column) and invasion assay Huh7-Elafin-U0126 (2^nd^ row, 6^th^ column)
- Fig. S7a, correct images used for Huh7-Neg-con at 0 h (1^st^ row, 5^th^ column), Huh7-Elafin-Erlotinib at 0 h (1^st^ row, 8^th^ column), Hep3B-Elafin-Erlotinib at 48 h (3^rd^ row, 4^th^ column), and Huh7-Elafin-Erlotinib at 48 h (3^rd^ row, 8^th^ column)

The authors provided the Journal with the original data files. The corrected figures are given here. The corrections do not have any effect on the final conclusions of the paper. The original article has been corrected.

**Fig. 2:**
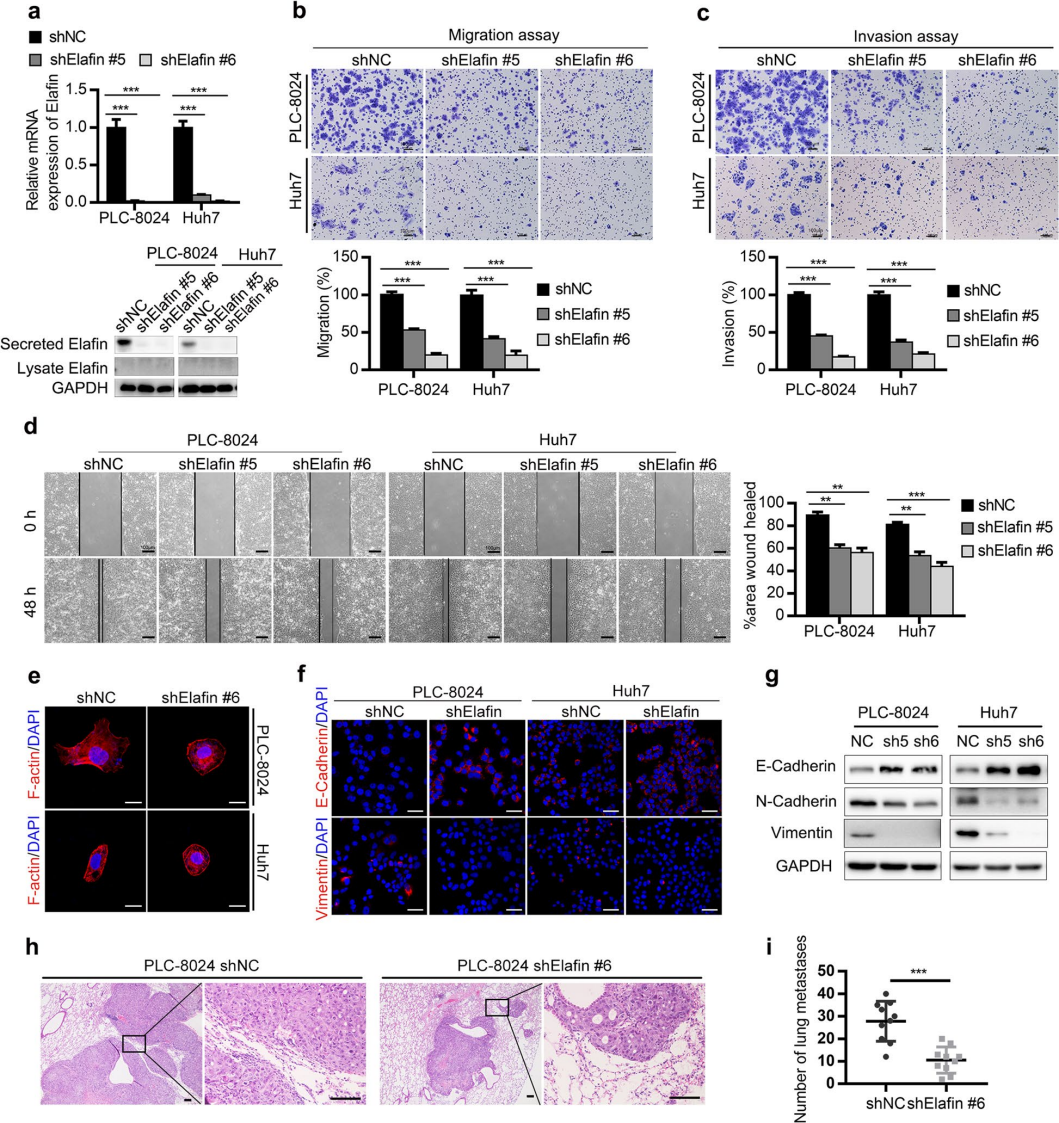
Knockdown of Elafin inhibits epithelial-mesenchymal transition (EMT) and metastasis of HCC cells in vitro and in vivo. **a** The efficiency of Elafin knockdown were measured by real-time PCR (top) and Western blotting (bottom) assays. *** *P* < 0.001. **b** Knockdown of Elafin resulted in suppressing migration of PLC-8024 and Huh7 cells. Scale bar, 100 μm. Statistical results are presented as mean ± SD (from triplicates), and significance is determined by Student t test (*** *P* < 0.001). **c** Knockdown of Elafin resulted in suppressing invasion of PLC-8024 and Huh7 cells. Scale bar, 100 μm. Statistical results are presented as mean ± SD (from triplicates) (*** *P* < 0.001). **d** Knockdown of Elafin impaired the scratch wound-healing ability of PLC-8024 and Huh7 cells. Scale bar, 100 μm. Statistical results at 48 h of scratch wound-healing assays are presented as mean ± SD (from triplicates) (** *P* < 0.01; *** *P* < 0.001). **e** Representative immunofluorescence images illustrating cytoskeleton of control and Elafin silencing cells. Scale bar, 20 μm. **f** Representative immunofluorescence images of EMT markers expression in Elafin silencing cells. Scale bar, 50 μm. **g** Expression of EMT markers mediated by Elafin silencing are shown by western boltting. **h** Down-regulation of Elafin significantly suppressed lung metastasis in nude mice model established by injection of indicated cells through the tail vein. Representative HE staining images are shown. Scale bar, 100 μm. **i** Lung metastasis nodules from the nude mice model are analyzed. *** *P* < 0.001

**Fig. 3:**
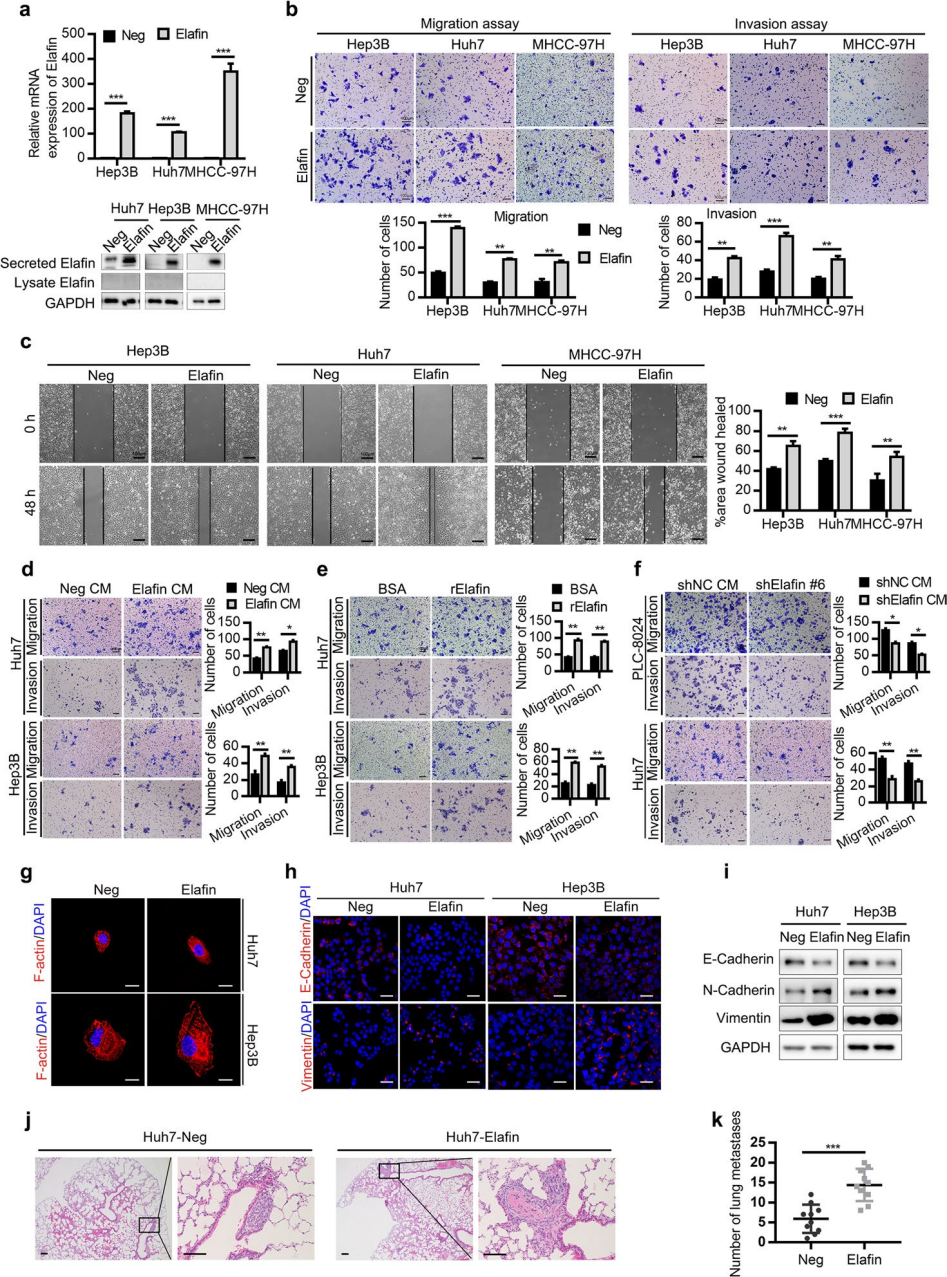
Overexpression of Elafin promotes EMT and metastasis of HCC cells in vitro and in vivo. **a** The efficiency of Elafin overexpression were measured by real-time PCR (top) and Western blotting (bottom) assays. *** *P* < 0.001. **b** Overexpression of Elafin enhanced migration and invasion of MHCC-97H, Huh7 and Hep3B cells. Scale bar, 100 μm. Statistical results are presented as mean ± SD (from triplicates), and significance is determined by Student t test (*** *P* < 0.001). **c** Overexpression of Elafin enhanced the scratch wound-healing ability of MHCC-97H, Hep3B and Huh7 cells. Scale bar, 100 μm. Statistical results at 48 h are presented as mean ± SD (from triplicates) (** *P* < 0.01; *** *P* < 0.001). **d** and **e** Concentrated Elafin-overexpression condition medium (Elafin CM) and commercial recombinant Elafin (rElafin, 10 μg/ml) promoted migration and invasion of wild-type HCC cells. Scale bar, 100 μm. Statistical results are presented as mean ± SD (from triplicates) (* *P* < 0.05; ** *P* < 0.01;*** *P* < 0.001). **f** The effects of concentrated Elafin-knockdown conditioned medium on HCC cells. Scale bar, 100 μm. Statistical results are presented as mean ± SD (from triplicates) (* *P* < 0.05; ** *P* < 0.01). **g** Representative immunofluorescence images illustrating cytoskeleton of control and Elafin overexpressing cells. Scale bar, 20 μm. **h** Representative immunofluorescence images of EMT markers expression in Elafin overexpressing cells. Scale bar, 50 μm. **i** Expression of EMT markers mediated by Elafin overexpression are shown by western boltting. **j** Overexpression of Elafin significantly enhanced lung metastasis in nude mice model established by injection of indicated cells through the tail vein. Representative HE staining images are shown. Scale bar, 100 μm. **k** Lung metastasis nodules from the nude mice model are analyzed. *** *P* < 0.001

**Fig. 5:**
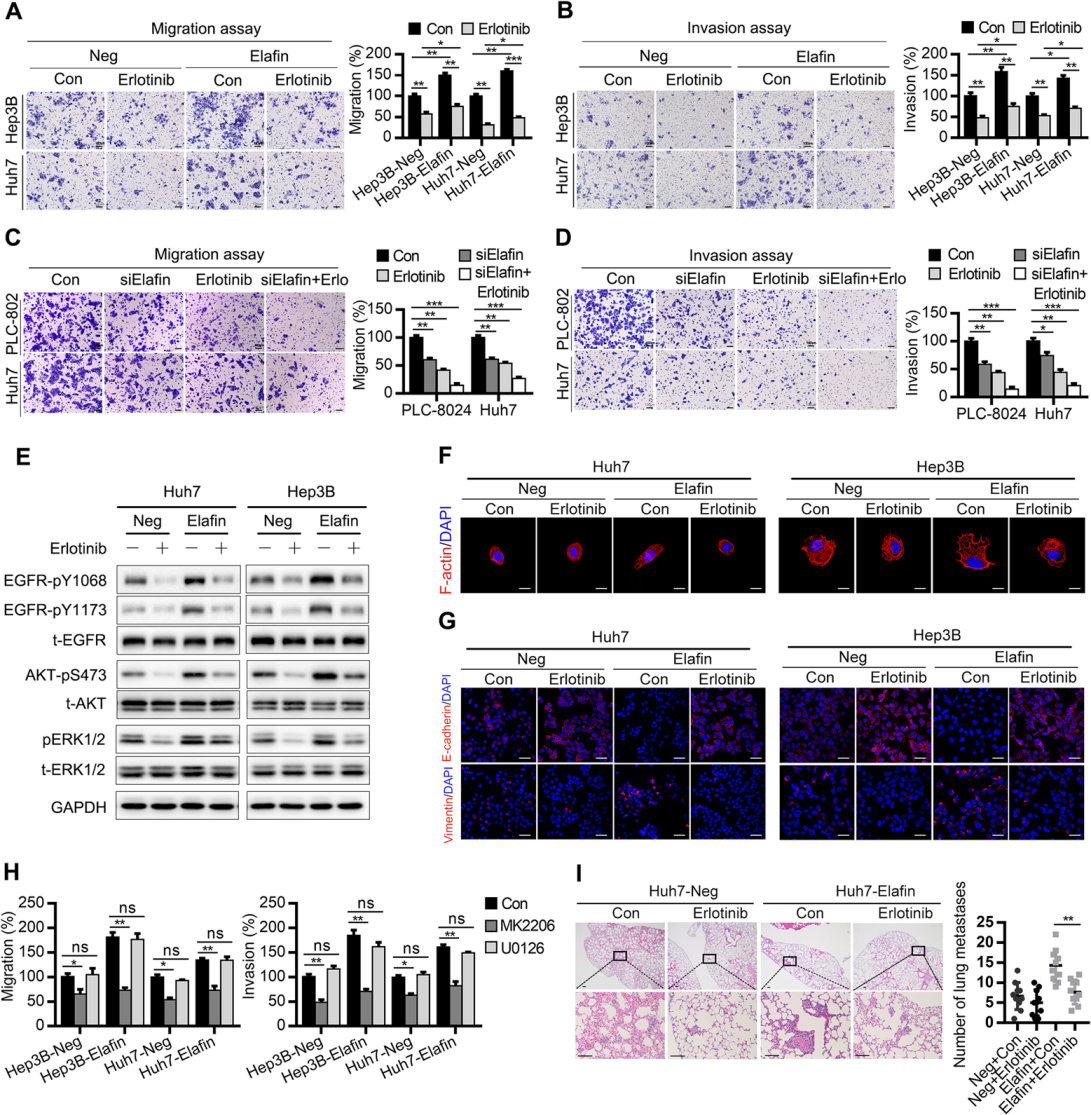
Elafin induces EMT and metastasis though EGFR/AKT signaling independently and attenuated the effect of erlotinib in HCC. **a** and **b** Inhibitor of EGFR impaired migration (**a**) and invasion (**b**) of Elafin-overexpressing HCC cells. Cells were treated with Erlotinib (3 μM) for 24 h and then were subjected to trans-well assays. Scale bar, 100 μm. Statistical results are presented as mean ± SD (from triplicates), and significance is determined by Student t test (* *P* < 0.05; ** *P* < 0.01; *** *P* < 0.001). **c** and **d** Down-regulated of Elafin enhanced the effect of Erlotinib on suppressing migration (**c**) and invasion (**d**) in HCC cells. After treated with siRNA of Elafin, erlotinib (3 μM), and both of them, indicated cells were conducted trans-well assays. Statistical results are presented as mean ± SD (* *P* < 0.05; ** *P* < 0.01; *** *P* < 0.001). **e** Erlotinib inhibited phosphorylation of EGFR and downstream signaling. Indicated cells were treated with erlotinib (3 μM) for 24 h and then performed western blotting. **f** Representative immunofluorescence images illustrating cytoskeleton of control and Elafin overexpressing cells after treated with Erlotinib (3 μM) for 24 h. Scale bar, 20 μm. **g** Representative immunofluorescence images of EMT markers expression in Elafin overexpressing cells after treated with Erlotinib (3 μM) for 24 h. Scale bar, 50 μm. **h** The effects of inhibitors of AKT and ERK on the migration and invasion of Elafinoverexpressing HCC cells. Cells were treated with MK2206 (1 μM) and U0126 (15 μM) for 12 h and then were subjected to transwell assays. Statistical results are presented as mean ± SD (ns, no significance; * *P* < 0.05; ** *P* < 0.01; *** *P* < 0.001). **i** Erlotinib impaired lung metastases of Elafin-overexpressing HCC cells in vivo. Representative HE staining images are shown. Scale bar, 100 μm. Quantification of lung metastasis nodules is analyzed. ** *P* < 0.01

## Supplementary Information

**Additional file 1.**

## References

[1] Wang C, Liao Y, He W (2021). Elafin promotes tumour metastasis and attenuates the anti-metastatic effects of erlotinib via binding to EGFR in hepatocellular carcinoma. J Exp Clin Cancer Res.

