# Supplementary material for: Elafin promotes tumour metastasis and attenuates the anti-metastatic effects of erlotinib via binding to EGFR in hepatocellular carcinoma
Source: J Exp Clin Cancer Res. 2021 Mar 26;40:113. doi: 10.1186/s13046-021-01904-y (PMC7995733; doi:10.1186/s13046-021-01904-y)
Supplement: Supplementary file 1 — Additional file 1. Supplementary Materials and Methods. [file 13046_2021_1904_MOESM1_ESM.zip › Supplementary Figure Legends_ESM.docx]

**Figure S1. The expression of Elafin in HCC patients and HCC cell lines.**

1. The expression levels of Elafin mRNA in 30 paired HCC and adjacent nontumor tissues were evaluated by quantitative real-time polymerase chain reaction.
2. Relative mRNA of Elafin levels in HCC cell lines and normal liver cell line MIHA. Statistical results are presented as mean±SD (from triplicates), and significance is determined by Student t test (ns, no significance; ** P < 0.01; *** P < 0.001).
3. Elafin protein was predominantly present in the condition medium instead of the whole cell lysate. Western blots assay was performed to identified the Elafin protein levels in whole cell lysate (top) and condition medium (bottom).

**Figure S2. Knockdown of Elafin has no effects on the proliferation of HCC cells.**

1. Cell growth of Elafin knockdown cells measured by CCK8 assays is shown. One thousand indicated cells were plated for CCK8 and measured the absorbance at 450nm for the following five days. Results were obtained from three independent assays. Statistical results are presented as mean±SD, and significance is determined by Student t test (ns, no significance).
2. Cell growth of Elafin knockdown cells was measured by colony formation assays. One thousand indicated cells were plated for colony formation assay, 10-14 days after then, the cells were fixed, stained, and photographed. Results were obtained from three independent assays. Statistical results are presented as mean±SD.

**Figure S3. Overexpression of Elafin has no effects on the proliferation of HCC cells.**

1. Cell growth of Elafin overexpression cells measured by CCK8 assays is shown. One thousand indicated cells were plated for CCK8 and measured the absorbance at 450nm for the following five days. Results were obtained from three independent assays. Statistical results are presented as mean±SD, and significance is determined by Student t test (ns, no significance).
2. Cell growth of Elafin overexpression cells was measured by colony formation assays. One thousand indicated cells were plated for colony formation assay, 10-14 days after then, the cells were fixed, stained, and photographed. Results were obtained from three independent assays. Statistical results are presented as mean±SD.

**Figure S4. Elafin induces EGFR phosphorylation and EMT of HCC cells.**

(A) rElafin induced EGFR phosphorylation. Huh7 cells were starved for 24h and then treated with BSA, rElafin (10µg/ml) and EGF (100ng/ml) for 30min, then were subjected to western blotting assay.

(B) rElafin stimulated HCC cells exhibiting an elongated and spinous cellular morphology as EGF. Huh7 cells were starved for 24h and then treated with BSA, rElafin (10µg/ml) and EGF (100ng/ml) for 30min, then immunofluorescence staining was performed to illustrate the cytoskeleton of the cells. Scale bar, 20µm.

**Figure S5. EGFR downstream pathways regulated by Elafin.**

Western blotting detection of the key markers of EGFR downstream signaling pathways in Elafin knockdown (A) and overexpressed (B) HCC cells.

**Figure S6. AKT signaling instead of ERK signaling independently contributes to the Elafin-induced HCC metastasis.**

1. Western blots were performed to identify the best concentration conditions of AKT inhibitors (MK2206).
2. Western blots were performed to identify the best concentration conditions of ERK inhibitors (U0126).
3. Migration assays (top) and invasion assays (bottom) were performed after Huh7 cells pretreated with AKT inhibitor (MK2206,1µM) and ERK inhibitor (U0126,15µM) for 12h.

**Figure S7. Erlotinib impaired the scratch wound-healing ability of Elafin-overexpression HCC cells.**

1. Cells were treated with Erlotinib (3µM) for 24h and then were subjected to scratch wound-healing assays. Scale bar, 100µm.
2. Quantitative data from (A), statistical results are presented as mean±SD (from triplicates), and significance is determined by Student *t* test (* P < 0.05; ** P < 0.01).

**Figure S8. The knockdown efficiency of siRNAs of Elafin in HCC cells.**

Huh7 cells were transiently transfected with various siRNAs of Elafin, after 48h post transfection, the mRNA expression of Elafin was detected by real-time PCR.
