# Supplementary material for: Elafin promotes tumour metastasis and attenuates the anti-metastatic effects of erlotinib via binding to EGFR in hepatocellular carcinoma
Source: J Exp Clin Cancer Res. 2021 Mar 26;40:113. doi: 10.1186/s13046-021-01904-y (PMC7995733; doi:10.1186/s13046-021-01904-y)
Supplement: Supplementary file 1 — Additional file 1. Supplementary Materials and Methods. [file 13046_2021_1904_MOESM1_ESM.zip › Supplementary Materials and Methods._ESM.docx]

**Supplementary Materials and Methods**

**Preparation and concentration of the condition medium (CM)**

Wild type and the stable HCC cells (3×10^6^) were plated in 100 mm culture dish and incubated for 48 hours in regular conditions. Subsequently, after removing the supernatant and washing with 1× phosphate-buffered saline (PBS), the cells were incubated with 10 ml serum-free DMEM for 24 hours. After then, conditional medium (CM) was collected and concentrated to a volume of 200 µL by ultrafiltration with Amicon Uitra-15 centrifugal filters (Merck Millipore, Cork, Ireland). Fifty µL of the concentrated condition medium was subjected to western blotting and the rest was stored in aliquots at -80℃ until used.

**RNA extraction and quantitative real-time PCR**

Total RNA was isolated from cell lines using Trizol reagent (Life Technologies, Carlsbad, CA) according to the manufacturer’s instructions. Complementary DNA was synthesized using random hexamers and superscript III (Invitrogen, California, USA) from 1µg of total RNA. Then, quantitative real-time PCR was performed on LightCycler 480 II detector (Roche, Basel, Switzerland) using SYBR Green PCR kit (Invitrogen, California, USA). All experiments were run in triplicate, and β-actin was used as control gene to normalize the gene expression levels. The used primers in this study were shown in **Table S5**.

**Western blotting assays**

The extracted whole cell protein lysates were prepared with cell lysis buffer (Cell Signaling Technology, Boston, USA) supplemented with protease inhibitor cocktail (Roche, Basel, Switzerland) and phosphatase inhibitor cocktail (Roche, Basel, Switzerland), when phosphorylated proteins were needed to detect. Then, the equal amounts of protein lysates were separated by SDS-polyacrylamide gels and transferred to polyvinylidene difluoride membranes (Merck Millipore, Cork, Ireland). After incubated with the indicated primary and secondary antibodies the bindings were detected using High-sig ECL western blotting kit (Tanon, shanghai, China).

**Cell proliferation and colony formation assays**

The cell proliferation was conducted with Cell Counting kit-8 (CCK-8; DojinDo, Japan) reagent. Briefly, one thousand cells were seeded in 96-well plates and cultured in DMED containing 10% FBS. Two hours after replaced the supernatant with fresh medium containing CCK-8 reagent in a 10:1 ratio, the relative cell density was measured by Biotek Epoch 2 machine (BioTek, Winooski, USA) at 450 nm. Six repeated wells were used for each experimental condition, and three independent experiments were performed.

For the colony formation assay, indicated stable cells with a number of 1000 were plated per well in 6-well plates in triplicate. Then the cells were incubated with DMEM containing 10% FBS and the medium was refreshed every 4 days. Fourteen days later, the cells were fixed in methanol and stained with 0.1% crystal violet. Images of the colonies were photographed and counted to contrast with each other.

***In vitro* migration and invasion assays and scratch wound healing assay**

Trans-well chambers (Costar, Kennebunk, USA) with polycarbonate membranes were used in migration assay, while chambers with matrigel (Costar, Kennebunk, USA) were used in invasion assay. For transwell assays, a number of 5×10^4^ to 1.5×10^5^ indicated cells were seeded into the upper chamber containing 200µL serum-free DMEM, at the same time, 800µL DMEM containing 10%FBS was added to the bottom chamber. After incubated for 8-12 hours, cells migrating to the lower surface of the upper chamber were fixed in methanol, stained with 0.1% crystal violet, and counted under the microscope. All experiments were triplicate and the migration and invasion cells were counted in four random optical fields of each chamber.

For scratch wound healing assay, 3×10^5^ cells were plated in six-well plates and incubated in the regular condition until the cells reached the full confluence of the plates. The wound was created by a sterile 100 µL pipette tip and detached cells were removed by PBS, and then the cells were incubated with serum-free DMEM for the indicated time. Images at 0, 24, and 48 h after scratching were taken, and images in the same region of each well were contrasted.

**Stable cell line construction and RNA interfering**

For Elafin knockdown cells, the lentivirus vectors containing the human short hairpin RNA Elafin (shElafin) or control shRNA were established by the Lenti-PacTM HIV Expression Packaging System (GeneCopoeia, Rockville, USA), according to the manufacturer’s instructions. The commercially available constructed lentivirus vectors which overexpress Elafin were purchased from GeneCopoeia. Elafin knockdown and overexpression cell sublines were constructed by transducting the indicated HCC cells using the concentrated viral supernatants or the purchased viral solution, together with 6 µg/ml polybrene (GeneCopoeia, Rockville, USA). Twelve hours after transduction, the medium was exchanged for fresh medium. Three days later, 1-2 µg/ml puromycin (Gibco; California, USA) was used to select the stably transfected cells for the following 3-5 days. After then, the selected stable transfected clones were validated by qRT-PCR and western blottings.

Small interfering RNA for Sp1, PI3 were purchased from Genepharma (Shanghai, China). Reverse transfection of small interfering RNA was performed with Lipofectamine-RNAiMAX (Invitrogen, Carlsbad, CA). After 24 hours, the supernatant was replaced with fresh medium and the down-regulation efficiency was identified by qRT-PCR and western blottings 48 hours after cotransfections. Targeting sequences were listed in the **Table S7**.

**Immunofluorescence staining (IF)**

Cells grown in 8-well BD Falcon cell culture slides (Millipore, MI, USA) were washed three times with ice-cold PBS and then were fixed with 4% paraformaldehyde (Jetway Biotech, Guangzhou, China) for 15 min. After permeabilizing with 0.2% Triton X-100 for 10 min at room temperature, the cells were blocked in goat serum (Zsbio, Beijing, China). Thereafter, the cells were incubated with anti-Vimentin, anti-E-cadherin, or anti-EGFR antibodies, as described in **Table S4**, at 4℃ overnight. Then, after washing with ice-cold PBS for three times, the cells were incubated with AlexaFluor488 or AlexaFluor594 goat anti-rabbit IgG (Invitrogen, California, USA) at room temperature for 1h avoiding lights. The nuclei staining was performed by DAPI (Beyotime, Shanghai, China) for 10 min, and then the stained cells were observed with a laser scanning confocal microscope. F-actin staining was performed using Texas Red-X Phalloidin (Invitrogen, Carlsbad, CA).
