# Supplementary material for: Elafin promotes tumour metastasis and attenuates the anti-metastatic effects of erlotinib via binding to EGFR in hepatocellular carcinoma
Source: J Exp Clin Cancer Res. 2021 Mar 26;40:113. doi: 10.1186/s13046-021-01904-y (PMC7995733; doi:10.1186/s13046-021-01904-y)
Supplement: Supplementary file 1 — Additional file 1. Supplementary Materials and Methods. [file 13046_2021_1904_MOESM1_ESM.zip › Supplementary Tables._ESM.docx]

Table S1. Demographic and clinical characteristics of the enrolled patients.

| Variable | High Elafin  (n = 240) | Low Elafin  (n = 138) | P Value |
| --- | --- | --- | --- |
| Gender (Male) | 215 (89.6) | 119(86.2) | 0.417 |
| age (y) | 53.00 [43.00, 61.00] | 51.00 [42.25, 59.75] | 0.561 |
| HBV infection (yes) | 221 (92.1) | 124 (89.9) | 0.583 |
| HCV infection (yes) | 3 (1.2) | 5 (3.6) | 0.147 |
| HBV-DNA (High) | 156 (65.0) | 83 (60.1) | 0.406 |
| ALT (U/L) | 39.05 [27.25, 57.07] | 34.45 [25.77, 49.08] | 0.090 |
| AST (U/L) | 37.30 [28.90, 56.30] | 35.05 [26.18, 49.27] | 0.065 |
| ALP (U/L) | 91.05 [72.38, 112.62] | 86.45 [73.32, 109.43] | 0.445 |
| ALB (g/L) | 41.85 [39.10, 44.23] | 42.50 [39.80, 44.30] | 0.331 |
| TBIL (µmol/L) | 12.80 [10.28, 17.60] | 12.85 [10.53, 16.90] | 0.803 |
| GGT (U/L) | 61.90 [39.42, 119.77] | 57.10 [36.60, 116.40] | 0.558 |
| AFP (ng/mL) | 175.20 [8.01, 2837.00] | 27.77 [6.62, 1130.00] | 0.035 |
| Resection margin (≥1/<1cm) | 28 (11.7) | 7 (5.1) | 0.052 |
| Cirrhosis (yes) | 191 (79.6) | 96 (69.6) | 0.039 |
| Multiple tumors (yes) | 75 (31.3) | 26 (18.8) | 0.012 |
| Tumor size (cm) | 5.50 [3.58, 8.50] | 6.00 [4.00, 9.00] | 0.440 |
| Tumor seed (yes) | 49 (20.4) | 26 (18.8) | 0.813 |
| adjacent Organ Invasion (yes) | 21 (8.7) | 4 (2.9) | 0.031 |
| Lymph node metastasis (yes) | 15 (6.2) | 9 (6.5) | 1.000 |
| Poor tumour differentiation (yes) | 27 (11.2) | 16 (11.6) | 1.000 |
| Microvascular invasion (yes) | 89 (37.1) | 50 (36.2) | 0.957 |
| Tumor capsule (no) | 113 (47.1) | 39 (28.3) | <0.001 |
| Tumor thrombus (yes) | 87 (36.3) | 33 (23.9) | 0.018 |

values are presented as the median (interquartile range) or n (%).

Abbreviation:

HBV: hepatitis B virus. HCV: hepatitis C virus. AFP: alpha-fetoprotein. ALB: albumin. TBIL: total bilirubin. ALT: alanine aminotransferase. AST: aspartate aminotransferase. ALP: alkaline phosphatase. GGT: γ-glutamyl transpeptidase.

Table S2. Univariate and multivariate cox regression analyses of the prognostic factors for overall survival.

| Variable | Univariate analysis | | Multivariate analysis | |
| --- | --- | --- | --- | --- |
|  | HR (95% CI) | P Value | HR (95% CI) | P Value |
| Gender (Male/Female) | 0.692 (0.37-1.3) | 0.242 |  |  |
| Age, y (≤60/>60) | 0.885 (0.6-1.3) | 0.537 |  |  |
| HBV infection (no/yes) | 0.881 (0.5-1.6) | 0.664 |  |  |
| HCV infection(no/yes) | 1.49 (0.47-4.7) | 0.495 |  |  |
| HBV-DNA(Normal/High) | 1.32 (0.92-1.9) | 0.135 |  |  |
| ALT, U/L (≤50/>50) | 1.7 (1.2-2.4) | 0.003 | 1.36(0.91-2.05) | 0.137 |
| AST, U/L (≤40/>40) | 2.11 (1.5-3) | < 0.001 | 1.34(0.87-2.06) | 0.171 |
| ALP, U/L (≤125/>125) | 0.968 (0.61-1.5) | 0.888 |  |  |
| ALB, g/L (≥35/<35) | 1.4 (0.73-2.7) | 0.310 |  |  |
| TBIL, µmol/L (≤17.1/>17.1) | 1.18 (0.8-1.7) | 0.401 |  |  |
| GGT, U/L (≤60/>60) | 1.86 (1.3-2.6) | < 0.001 | 1.34(0.91-1.99) | 0.135 |
| AFP, ng/mL (≤200/>200) | 1.96 (1.4-2.8) | < 0.001 | 1.56(1.09-2.23) | 0.014 |
| Resection margin (≥1/<1cm) | 1.1 (0.59-2) | 0.773 |  |  |
| Cirrhosis(no/yes) | 0.778 (0.53-1.1) | 0.195 |  |  |
| Multiple tumors (no/yes) | 1.81 (1.2-2.7) | 0.002 | 4.92(0.56-42.6) | 0.148 |
| Tumor size (≤5/>5) | 1.67 (1.2-2.4) | 0.004 | 1.22(0.83-1.81) | 0.301 |
| Tumor seed (no/yes) | 1.75 (1.2-2.6) | 0.005 | 3.12(1.52-2.75) | 0.300 |
| Adjacent Organ Invasion (no/yes) | 2.3 (1.2-4.5) | 0.016 | 1.23(0.58-2.62) | 0.576 |
| Poor tumour differentiation (no/yes) | 1.29 (0.78-2.2) | 0.321 |  |  |
| Microvascular invasion (no/yes) | 1.28 (0.91-1.8) | 0.161 |  |  |
| Tumor capsule (yes/no) | 1.07 (0.75-1.5) | 0.692 |  |  |
| Tumor thrombus (no/yes) | 2.21 (1.6-3.1) | < 0.001 | 1.91(1.32-2.76) | < 0.001 |
| Elafin (Low/High) | 1.86 (1.3-2.7) | 0.001 | 1.73 (1.17-2.55) | 0.005 |

Abbreviation:

HR: hazard ratio. CI: confidence interval. HBV: hepatitis B virus. HCV: hepatitis C virus. AFP: alpha-fetoprotein. ALB: albumin. TBIL: total bilirubin. ALT: alanine aminotransferase. AST: aspartate aminotransferase. ALP: alkaline phosphatase. GGT: γ-glutamyl transpeptidase.

Table S3. Univariate and multivariate cox regression analyses of the prognostic factors for recurrence-free survival.

| Variables | Univariable analysis | | Multivariable analysis | |
| --- | --- | --- | --- | --- |
|  | HR (95% CI) | P Value | HR (95% CI) | P Value |
| Gender (Male/Female) | 0.836 (0.55-1.3) | 0.399 |  |  |
| Age, y (≤60/>60) | 0.89 (0.66-1.2) | 0.434 |  |  |
| HBV infection (no/yes) | 1.42 (0.86-2.3) | 0.166 |  |  |
| HCV infection (no/yes) | 2.24 (1.1-4.5) | 0.025 |  |  |
| HBV-DNA(Normal/High) | 1.39 (1.1-1.8) | 0.018 | 1.35(1.00-1.82) | 0.047 |
| ALT, U/L (≤50/>50) | 1.31 (0.99-1.7) | 0.061 | 0.94(0.67-1.34) | 0.769 |
| AST, U/L (≤40/>40) | 1.76 (1.4-2.3) | < 0.001 | 1.43(1.01-2.01) | 0.039 |
| ALP, U/L (≤125/>125) | 1.2 (0.86-1.7) | 0.281 |  |  |
| ALB, g/L (≥35/<35) | 1.28 (0.77-2.1) | 0.346 |  |  |
| TBIL, µmol/L (≤17.1/>17.1) | 0.985 (0.73-1.3) | 0.919 |  |  |
| GGT, U/L (≤60/>60) | 1.5 (1.2-1.9) | 0.002 | 1.05(0.78-1.42) | 0.715 |
| AFP, ng/mL (≤200/>200) | 1.58 (1.2-2) | < 0.001 | 1.20(0.91-1.61) | 0.192 |
| Resection margin (≥1/<1cm) | 1.13 (0.72-1.8) | 0.605 |  |  |
| Cirrhosis(no/yes) | 1.09 (0.8-1.5) | 0.597 |  |  |
| Multiple tumors (no/yes) | 1.54 (1.1-2.1) | 0.005 | 2.97(0.35-24.7) | 0.313 |
| Tumor size (≤5/>5) | 1.51 (1.2-2) | 0.002 | 1.37(1.01-1.85) | 0.037 |
| Tumor seed (no/yes) | 1.52 (1.1-2.1) | 0.007 | 2.39(1.2-3.44) | 0.421 |
| Adjacent Organ Invasion (no/yes) | 1.74 (0.95-3.2) | 0.073 | 1.14(0.59-2.19) | 0.690 |
| Poor tumour differentiation (no/yes) | 1.15 (0.78-1.7) | 0.479 |  |  |
| Microvascular invasion (no/yes) | 1.15 (0.88-1.5) | 0.305 |  |  |
| Tumor capsule (yes/no) | 1.3 (1-1.7) | 0.051 | 1.47(1.12-1.95) | 0.005 |
| Tumor thrombus (no/yes) | 1.89 (1.4-2.5) | < 0.001 | 1.68(1.25-2.25) | < 0.001 |
| Elafin (Low/High) | 1.92 (1.2-3.1) | 0.006 | 2.09(1.27-3.43) | 0.003 |

Abbreviation:

HR: hazard ratio. CI: confidence interval. HBV: hepatitis B virus. HCV: hepatitis C virus. AFP: alpha-fetoprotein. ALB: albumin. TBIL: total bilirubin. ALT: alanine aminotransferase. AST: aspartate aminotransferase. ALP: alkaline phosphatase. GGT: γ-glutamyl transpeptidase.

Table S4. Antibodies included in the study

| Assays | Antibodies |
| --- | --- |
| western blotting assays | rabbit anti-Elafin (ab46774, abcam, 1:500); rabbit anti-E-Cadherin (610181, BD Biosciences, 1:1000); rabbit anti-N-Cadherin (#4061p, Cell Signaling Technology, 1:1000); rabbit anti-Vimentin (#5741, Cell Signaling Technology, 1:1000); mouse anti-Fibronectin (610077, BD Biosciences, 1:2000); rabbit anti-ZEB1 (HPA027524, Sigma-Aldrich, 1:2000); rabbit anti-Slug (#9585s, Cell Signaling Technology, 1:500); rabbit anti-SNAIL (#3879s, Cell Signaling Technology, 1:500); mouse anti-GAPDH (60004-1-Ig, Proteintech, 1:2000); rabbit anti-EGFR-pY1068 (#3777s, Cell Signaling Technology, 1:1000), rabbit anti-EGFR-pY1173 (#4407s, Cell Signaling Technology, 1:1000), rabbit anti-EGFR (#4267s, Cell Signaling Technology, 1:1000), rabbit anti-AKT-pS473 (#4060s, Cell Signaling Technology, 1:1000), rabbit anti-pan-AKT (#4685s, Cell Signaling Technology, 1:1000), rabbit anti-p-ERK1/2 (#4370p, Cell Signaling Technology, 1:2000), rabbit anti-ERK1/2 (#4695s, Cell Signaling Technology, 1:2000), rabbit anti-p-SAPK/JNK (#4668s, Cell Signaling Technology, 1:500), rabbit anti-SAPK/JNK (#9258p, Cell Signaling Technology, 1:1000), rabbit anti-p-MAPK38 (#4511s, Cell Signaling Technology, 1:1000), rabbit anti-MAPK38 (#9212s, Cell Signaling Technology, 1:1000), rabbit anti-p-GSK-3β (#9323p, Cell Signaling Technology, 1:1000), rabbit anti- GSK-3β (22104-1-AP, Proteintech, 1:1000), rabbit anti-p-STAT3 (#9134s, Cell Signaling Technology, 1:1000), rabbit anti-STAT3 (#9139p, Cell Signaling Technology, 1:1000), rabbit anti-Sp1 (#9389s, Cell Signaling Technology, 1:1000). |
| immunohistochemical staining (IHC) | rabbit anti-Elafin (ab46774, abcam, 1:800); rabbit anti-Vimentin (#5741, Cell Signaling Technology, 1:1000); rabbit anti-Sp1 (#9389s, Cell Signaling Technology, 1:800); rabbit anti-AKT-pS473 (#4060s, Cell Signaling Technology, 1:500). |
| immunofluorescence (IF) | rabbit anti-Vimentin (#5741, Cell Signaling Technology, 1:500); rabbit anti-N-Cadherin (#4061p, Cell Signaling Technology, 1:400); rabbit anti-EGFR (#4267s, Cell Signaling Technology, 1:400). |
| Co-IP assay | rabbit anti-EGFR (#4267s, Cell Signaling Technology, 1:100); goat anti-Elafin (PA5-47194, ThermoFisher, 25µg/ml);  Rabbit IgG (Proteintech). |
| CHIP assay | rabbit anti-Sp1 (#9389s, Cell Signaling Technology, 1:100); Rabbit IgG (Proteintech) |

Table S5. The primers used in present study.

| Primer names | sequences |
| --- | --- |
| PI3 | Forward (5’-3’): CGCTGCTTGAAAGATACTGACTG  Reverse (5’-3’): ACGGCACAGGTGCAGCAAGGA |
| ACTB | Forward (5’-3’): CACCATTGGCAATGAGCGGTTC  Reverse (5’-3’): AGGTCTTTGCGGATGTCCACGT |
| Primer in CHIP assay | Forward (5’-3’): AGGAGGGAAACCTGGCTGTG  Reverse (5’-3’): TGAGTAATGAAAGGGGAAAACG |

Table S6. Elafin-interacting membrane proteins identified by mass spectrometry are listed.

| N | Unused | %Cov (95) | Accession # | Peptides (95%) |
| --- | --- | --- | --- | --- |
| 1 | 117.04 | 28.8 | sp\|Q13813\|SPTN1_HUMAN | 54 |
| 2 | 113.69 | 32.9 | sp\|P35579\|MYH9_HUMAN | 71 |
| 3 | 107.87 | 27.9 | sp\|Q01082\|SPTB2_HUMAN | 52 |
| 4 | 69.62 | 24.5 | sp\|P35580\|MYH10_HUMAN | 39 |
| 5 | 64.4 | 42.9 | sp\|P04264\|K2C1_HUMAN | 48 |
| 6 | 48.87 | 53.8 | sp\|P35908\|K22E_HUMAN | 28 |
| 7 | 46.35 | 38.9 | sp\|P13645\|K1C10_HUMAN | 30 |
| 8 | 39.68 | 43.2 | sp\|P35527\|K1C9_HUMAN | 25 |
| 9 | 33 | 12.4 | sp\|Q00610\|CLH1_HUMAN | 16 |
| 10 | 32.34 | 39.4 | sp\|P09493\|TPM1_HUMAN | 18 |
| 11 | 30.58 | 53.3 | sp\|P63261\|ACTG_HUMAN | 44 |
| 12 | 29.49 | 11.8 | sp\|Q8WWI1\|LMO7_HUMAN | 14 |
| 13 | 28.97 | 17.5 | sp\|P09874\|PARP1_HUMAN | 13 |
| 14 | 28.72 | 23 | sp\|P00533\|EGFR_HUMAN | 37 |
| 15 | 28.46 | 65.2 | sp\|P09211\|GSTP1_HUMAN | 59 |
| 16 | 23.52 | 7.9 | sp\|Q7Z406\|MYH14_HUMAN | 14 |
| 17 | 22.95 | 61.2 | sp\|P62805\|H4_HUMAN | 20 |
| 18 | 18.25 | 25.7 | sp\|P16401\|H15_HUMAN | 17 |
| 19 | 16.74 | 19.2 | sp\|P13647\|K2C5_HUMAN | 11 |
| 20 | 16.27 | 51.5 | sp\|P19105\|ML12A_HUMAN | 9 |
| 21 | 14.67 | 58.1 | sp\|Q9BTM1\|H2AJ_HUMAN | 13 |
| 22 | 14.45 | 27.7 | sp\|P16403\|H12_HUMAN | 15 |
| 23 | 13.83 | 39.9 | sp\|P67936\|TPM4_HUMAN | 16 |
| 24 | 12.25 | 13 | sp\|P11142\|HSP7C_HUMAN | 6 |
| 25 | 11.87 | 45 | sp\|P60660\|MYL6_HUMAN | 10 |
| 26 | 10.65 | 6.5 | sp\|Q9Y2D5\|AKAP2_HUMAN | 5 |
| 27 | 10.63 | 15.9 | sp\|P05787\|K2C8_HUMAN | 7 |
| 28 | 9.97 | 35.7 | sp\|Q99880\|H2B1L_HUMAN | 22 |
| 29 | 8.95 | 28.1 | sp\|P62987\|RL40_HUMAN | 5 |
| 30 | 8.71 | 24.5 | sp\|P06748\|NPM_HUMAN | 4 |
| 31 | 8.49 | 7.1 | sp\|P11940\|PABP1_HUMAN | 5 |
| 32 | 8.19 | 19.9 | sp\|Q71DI3\|H32_HUMAN | 6 |
| 33 | 8.05 | 22.7 | sp\|P23284\|PPIB_HUMAN | 6 |
| 34 | 8.02 | 31 | sp\|P39019\|RS19_HUMAN | 4 |
| 35 | 7.82 | 17.7 | sp\|O75367\|H2AY_HUMAN | 4 |
| 36 | 7.69 | 15.1 | sp\|P38159\|RBMX_HUMAN | 5 |
| 37 | 7.66 | 25.3 | sp\|P29966\|MARCS_HUMAN | 4 |
| 38 | 7.61 | 27 | sp\|P62269\|RS18_HUMAN | 5 |
| 39 | 7.52 | 10.9 | sp\|Q16643\|DREB_HUMAN | 4 |
| 40 | 7.44 | 16 | sp\|P07910\|HNRPC_HUMAN | 4 |
| 41 | 7.42 | 21.3 | sp\|P02538\|K2C6A_HUMAN | 10 |
| 42 | 7.2 | 6.5 | sp\|O43707\|ACTN4_HUMAN | 5 |
| 43 | 7.13 | 11.3 | sp\|P02768\|ALBU_HUMAN | 9 |
| 44 | 7.02 | 5.3 | sp\|Q9UHB6\|LIMA1_HUMAN | 3 |
| 45 | 6.74 | 7.5 | sp\|P23246\|SFPQ_HUMAN | 4 |
| 46 | 6.6 | 26.5 | sp\|Q02539\|H11_HUMAN | 8 |
| 47 | 6.22 | 41.1 | sp\|P17096\|HMGA1_HUMAN | 3 |
| 48 | 6.21 | 19.4 | sp\|P09496\|CLCA_HUMAN | 4 |
| 49 | 6.08 | 22.7 | sp\|P81605\|DCD_HUMAN | 3 |
| 50 | 6.07 | 3.6 | sp\|Q9UM54\|MYO6_HUMAN | 3 |
| 51 | 6.05 | 5.8 | sp\|Q9Y608\|LRRF2_HUMAN | 3 |
| 52 | 6.05 | 9.9 | sp\|P07437\|TBB5_HUMAN | 3 |
| 53 | 6.03 | 12.1 | sp\|Q96C19\|EFHD2_HUMAN | 3 |
| 54 | 6 | 29.4 | sp\|P52926\|HMGA2_HUMAN | 3 |
| 55 | 5.7 | 30.9 | sp\|P62158\|CALM_HUMAN | 4 |
| 56 | 5.55 | 33.7 | sp\|P06753\|TPM3_HUMAN | 15 |
| 57 | 5.53 | 5.6 | sp\|P19338\|NUCL_HUMAN | 3 |
| 58 | 5.27 | 15.2 | sp\|P26373\|RL13_HUMAN | 3 |
| 59 | 5.17 | 33.9 | sp\|P05387\|RLA2_HUMAN | 4 |
| 60 | 4.67 | 31.3 | sp\|Q71UI9\|H2AV_HUMAN | 5 |
| 61 | 4.66 | 1.6 | sp\|Q92614\|MY18A_HUMAN | 2 |
| 62 | 4.64 | 7.1 | sp\|P22626\|ROA2_HUMAN | 3 |
| 63 | 4.48 | 10.5 | sp\|Q9Y3Y2\|CHTOP_HUMAN | 2 |
| 64 | 4.38 | 15.1 | sp\|Q9NYL9\|TMOD3_HUMAN | 3 |
| 65 | 4.11 | 5.4 | sp\|Q9NZI8\|IF2B1_HUMAN | 2 |
| 66 | 4.05 | 6.8 | sp\|P35637\|FUS_HUMAN | 2 |
| 67 | 4.04 | 15.9 | sp\|P62277\|RS13_HUMAN | 2 |
| 68 | 4.01 | 6.3 | sp\|P61978\|HNRPK_HUMAN | 2 |
| 69 | 4 | 52.5 | sp\|P16104\|H2AX_HUMAN | 12 |
| 70 | 4 | 1.9 | sp\|Q9P2E9\|RRBP1_HUMAN | 2 |
| 71 | 4 | 20.3 | sp\|P62847\|RS24_HUMAN | 2 |
| 72 | 4 | 7.4 | sp\|P55795\|HNRH2_HUMAN | 2 |
| 73 | 4 | 6.2 | sp\|Q14103\|HNRPD_HUMAN | 2 |
| 74 | 4 | 17.8 | sp\|P83916\|CBX1_HUMAN | 2 |
| 75 | 4 | 15.1 | sp\|P62318\|SMD3_HUMAN | 2 |
| 76 | 4 | 17.5 | sp\|P62081\|RS7_HUMAN | 2 |
| 77 | 4 | 9.1 | sp\|P23396\|RS3_HUMAN | 2 |
| 78 | 4 | 7.7 | sp\|P07355\|ANXA2_HUMAN | 2 |
| 79 | 4 | 11.6 | sp\|P04406\|G3P_HUMAN | 2 |
| 80 | 3.88 | 5.1 | sp\|P21589\|5NTD_HUMAN | 2 |
| 81 | 3.62 | 57.7 | sp\|Q93077\|H2A1C_HUMAN | 12 |
| 82 | 3.58 | 2.3 | sp\|Q9Y2W1\|TR150_HUMAN | 2 |
| 83 | 3.48 | 2.4 | sp\|Q9UDY2\|ZO2_HUMAN | 2 |
| 84 | 3.35 | 35.7 | sp\|Q16778\|H2B2E_HUMAN | 21 |
| 85 | 3.27 | 7.1 | sp\|P38919\|IF4A3_HUMAN | 2 |
| 86 | 3.25 | 3.8 | sp\|P38646\|GRP75_HUMAN | 2 |
| 87 | 2.86 | 13.8 | sp\|Q07020\|RL18_HUMAN | 2 |
| 88 | 2.7 | 5 | sp\|Q07065\|CKAP4_HUMAN | 2 |
| 89 | 2.68 | 7.2 | sp\|P06576\|ATPB_HUMAN | 3 |
| 90 | 2.59 | 18.3 | sp\|P84090\|ERH_HUMAN | 2 |
| 91 | 2.46 | 2.4 | sp\|P25705\|ATPA_HUMAN | 1 |
| 92 | 2.4 | 2.7 | sp\|P09651\|ROA1_HUMAN | 1 |
| 93 | 2.4 | 1 | sp\|O14974\|MYPT1_HUMAN | 1 |
| 94 | 2.39 | 4 | sp\|Q07955\|SRSF1_HUMAN | 1 |
| 95 | 2.38 | 4 | sp\|P11021\|GRP78_HUMAN | 2 |
| 96 | 2.3 | 5.1 | sp\|P83731\|RL24_HUMAN | 1 |
| 97 | 2.28 | 38.7 | sp\|P07951\|TPM2_HUMAN | 18 |
| 98 | 2.24 | 4 | sp\|Q99623\|PHB2_HUMAN | 1 |
| 99 | 2.2 | 3.3 | sp\|P37837\|TALDO_HUMAN | 1 |
| 100 | 2.15 | 7.7 | sp\|P49006\|MRP_HUMAN | 1 |
| 101 | 2.13 | 7.6 | sp\|P62316\|SMD2_HUMAN | 1 |
| 102 | 2.12 | 5.9 | sp\|P61313\|RL15_HUMAN | 1 |
| 103 | 2.11 | 1.7 | sp\|P00966\|ASSY_HUMAN | 1 |
| 104 | 2.08 | 1.3 | sp\|O94832\|MYO1D_HUMAN | 1 |
| 105 | 2.08 | 4.3 | sp\|P62917\|RL8_HUMAN | 1 |
| 106 | 2.07 | 6.8 | sp\|P45973\|CBX5_HUMAN | 1 |
| 107 | 2.07 | 3.2 | sp\|O75475\|PSIP1_HUMAN | 1 |
| 108 | 2.06 | 0.9 | sp\|O95782\|AP2A1_HUMAN | 1 |
| 109 | 2.05 | 7.2 | sp\|P62899\|RL31_HUMAN | 1 |
| 110 | 2.04 | 5.5 | sp\|P46783\|RS10_HUMAN | 1 |
| 111 | 2.03 | 2.6 | sp\|P51991\|ROA3_HUMAN | 1 |
| 112 | 2.02 | 13.5 | sp\|O75531\|BAF_HUMAN | 1 |
| 113 | 2.02 | 1.8 | sp\|O00571\|DDX3X_HUMAN | 1 |
| 114 | 2.02 | 9.4 | sp\|P47914\|RL29_HUMAN | 1 |
| 115 | 2.02 | 1.4 | sp\|O43795\|MYO1B_HUMAN | 1 |
| 116 | 2.01 | 28.1 | sp\|P68133\|ACTS_HUMAN | 19 |
| 117 | 2.01 | 1.6 | sp\|Q8WXW3\|PIBF1_HUMAN | 2 |
| 118 | 2.01 | 0.8 | sp\|P15924\|DESP_HUMAN | 1 |
| 119 | 2.01 | 1.2 | sp\|P13010\|XRCC5_HUMAN | 1 |
| 120 | 2.01 | 2.2 | sp\|Q93084\|AT2A3_HUMAN | 1 |
| 121 | 2.01 | 8.1 | sp\|P61626\|LYSC_HUMAN | 1 |
| 122 | 2 | 18.3 | sp\|Q04695\|K1C17_HUMAN | 8 |
| 123 | 2 | 24.7 | sp\|P10412\|H14_HUMAN | 11 |
| 124 | 2 | 13.9 | sp\|P14649\|MYL6B_HUMAN | 2 |
| 125 | 2 | 2.2 | sp\|Q13310\|PABP4_HUMAN | 1 |
| 126 | 2 | 5.7 | sp\|P13804\|ETFA_HUMAN | 1 |
| 127 | 2 | 1.6 | sp\|Q9Y4W6\|AFG32_HUMAN | 1 |
| 128 | 2 | 0.4 | sp\|Q92616\|GCN1_HUMAN | 1 |
| 129 | 2 | 8.3 | sp\|P62750\|RL23A_HUMAN | 1 |
| 130 | 2 | 10.2 | sp\|P35268\|RL22_HUMAN | 1 |
| 131 | 2 | 0.7 | sp\|P31327\|CPSM_HUMAN | 1 |
| 132 | 2 | 0.8 | sp\|P11498\|PYC_HUMAN | 1 |
| 133 | 2 | 6.3 | sp\|Q9Y5S9\|RBM8A_HUMAN | 1 |
| 134 | 2 | 2 | sp\|Q9UNP9\|PPIE_HUMAN | 1 |
| 135 | 2 | 0.8 | sp\|Q9UBN4\|TRPC4_HUMAN | 1 |
| 136 | 2 | 1.1 | sp\|Q9NY28\|GALT8_HUMAN | 1 |
| 137 | 2 | 0.8 | sp\|Q9HCJ5\|ZSWM6_HUMAN | 1 |
| 138 | 2 | 1.8 | sp\|Q9BWU1\|CDK19_HUMAN | 1 |
| 139 | 2 | 2.2 | sp\|Q9BQE3\|TBA1C_HUMAN | 1 |
| 140 | 2 | 5.5 | sp\|Q96HU8\|DIRA2_HUMAN | 1 |
| 141 | 2 | 1.2 | sp\|Q92611\|EDEM1_HUMAN | 1 |
| 142 | 2 | 4.1 | sp\|Q8NGC8\|O11H7_HUMAN | 1 |
| 143 | 2 | 1.3 | sp\|Q8N6Q8\|MET25_HUMAN | 1 |
| 144 | 2 | 2.8 | sp\|Q86UD0\|SAPC2_HUMAN | 1 |
| 145 | 2 | 4.7 | sp\|Q7Z7K6\|CENPV_HUMAN | 1 |
| 146 | 2 | 2.2 | sp\|Q6UWY5\|OLFL1_HUMAN | 1 |
| 147 | 2 | 0.5 | sp\|Q5D862\|FILA2_HUMAN | 1 |
| 148 | 2 | 3.2 | sp\|Q13595\|TRA2A_HUMAN | 1 |
| 149 | 2 | 7.9 | sp\|P62913\|RL11_HUMAN | 1 |
| 150 | 2 | 10.4 | sp\|P62888\|RL30_HUMAN | 1 |
| 151 | 2 | 10.9 | sp\|P62314\|SMD1_HUMAN | 1 |
| 152 | 2 | 19.6 | sp\|P61513\|RL37A_HUMAN | 1 |
| 153 | 2 | 7.4 | sp\|P46776\|RL27A_HUMAN | 1 |
| 154 | 2 | 10.3 | sp\|P37108\|SRP14_HUMAN | 1 |
| 155 | 2 | 4.4 | sp\|P35232\|PHB_HUMAN | 1 |
| 156 | 2 | 0.8 | sp\|P21399\|ACOC_HUMAN | 1 |
| 157 | 2 | 6.8 | sp\|P12273\|PIP_HUMAN | 1 |
| 158 | 2 | 14 | sp\|P05386\|RLA1_HUMAN | 1 |
| 159 | 2 | 17 | sp\|P01834\|IGKC_HUMAN | 1 |
| 160 | 2 | 12.2 | sp\|P01040\|CYTA_HUMAN | 1 |
| 161 | 2 | 1.9 | sp\|O95747\|OXSR1_HUMAN | 1 |
| 162 | 2 | 1.6 | sp\|O94925\|GLSK_HUMAN | 1 |
| 163 | 2 | 2.1 | sp\|O76031\|CLPX_HUMAN | 1 |
| 164 | 2 | 1 | sp\|O15534\|PER1_HUMAN | 1 |
| 165 | 2 | 2.3 | sp\|O15105\|SMAD7_HUMAN | 1 |
| 166 | 2 | 1.7 | sp\|A2RTX5\|SYTC2_HUMAN | 1 |
| 167 | 1.95 | 13 | sp\|P05114\|HMGN1_HUMAN | 1 |
| 168 | 1.88 | 7.1 | sp\|P62829\|RL23_HUMAN | 1 |
| 169 | 1.73 | 3.6 | sp\|Q15424\|SAFB1_HUMAN | 2 |
| 170 | 1.71 | 1.5 | sp\|O00159\|MYO1C_HUMAN | 1 |
| 171 | 1.68 | 18.6 | sp\|P08779\|K1C16_HUMAN | 9 |
| 172 | 1.67 | 3.7 | sp\|P05783\|K1C18_HUMAN | 2 |
| 173 | 1.51 | 1.5 | sp\|Q05682\|CALD1_HUMAN | 1 |
| 174 | 1.42 | 12.2 | sp\|P07477\|TRY1_HUMAN | 2 |
| 175 | 1.37 | 9.3 | sp\|P67809\|YBOX1_HUMAN | 1 |

| Name | Sense strand/sense primer (5’-3’) | Antisense strand/antisense primer (5’-3’) |
| --- | --- | --- |
| siRNA dupleses | | |
| Si-PI3 | GCCAAGAAGUGCUGUGAAATT | UUUCACAGCACUUCUUGGCTT |
| Si-Sp1 | AAUGAGAACAGCAACAACUCC | GGAGUUGUUGCUGUUCUCAUU |
| NC | UUCUCCGAACGUGUCACGUTT | ACGUGACACGUUCGGAGAATT |

Table S7. Sequences of RNA Oligonucleotides.

Table S8. Predicted TF binding sites in the putative promoter regions of has-PI3 in JASPAR.

| Matrix ID | Name | Score | Relative score | Start | End | Strand | Predicted site sequence |
| --- | --- | --- | --- | --- | --- | --- | --- |
| MA0079.3 | Sp1 | 13.3712 | 0.949365332244 | 1647 | 1657 | － | TCCCCACCCCT |
| MA0079.3 | Sp1 | 12.6441 | 0.940217313184 | 2004 | 2014 | ＋ | TCTCCACCCCC |
| MA0079.3 | Sp1 | 10.928 | 0.918627065772 | 625 | 635 | － | CCTCCACCTCC |
