# Supplementary figures and images for: Elafin promotes tumour metastasis and attenuates the anti-metastatic effects of erlotinib via binding to EGFR in hepatocellular carcinoma
Source: J Exp Clin Cancer Res. 2021 Mar 26;40:113. doi: 10.1186/s13046-021-01904-y (PMC7995733; doi:10.1186/s13046-021-01904-y)

**Figure S1.**

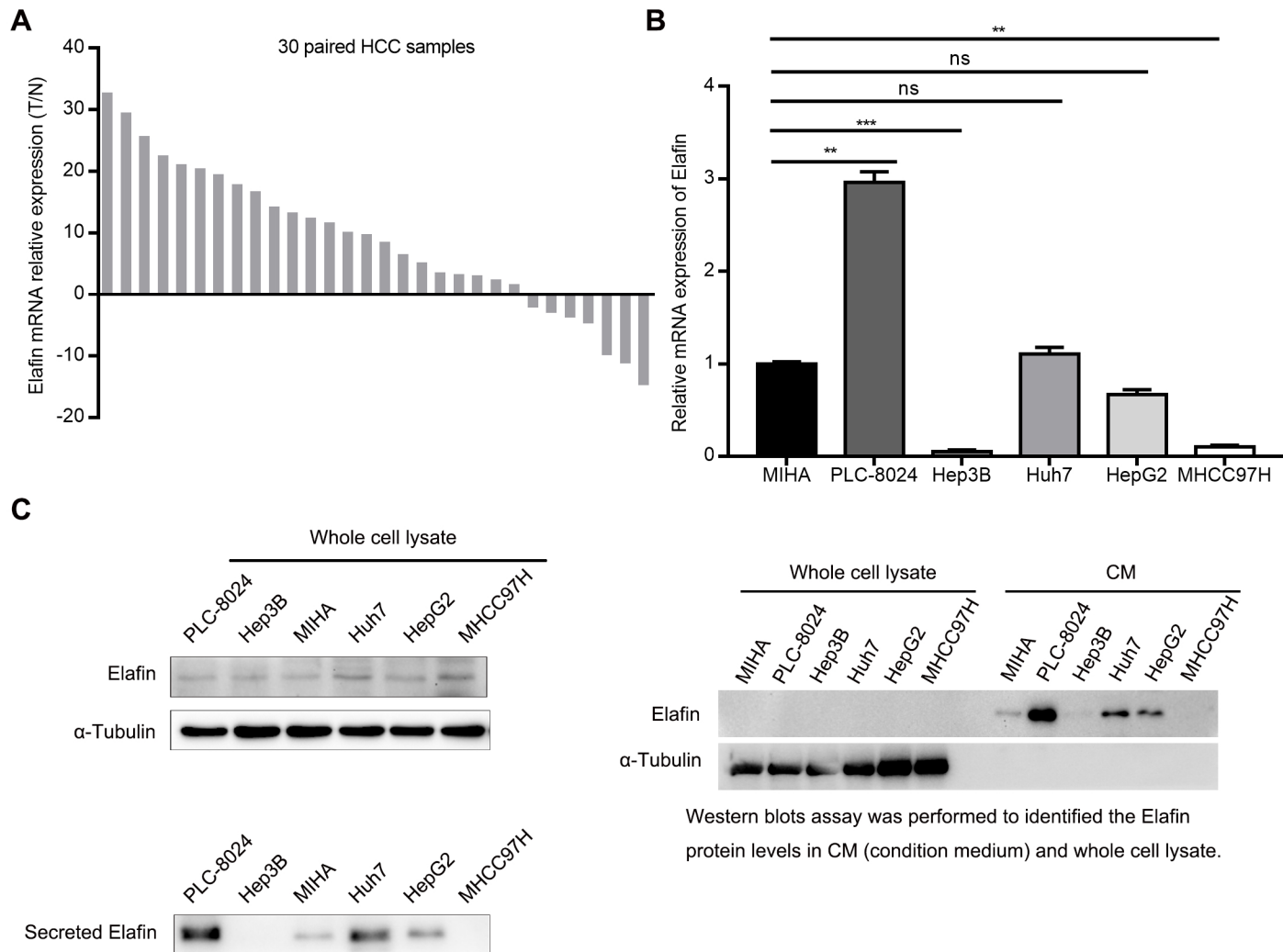

**Figure S2.**

**A**

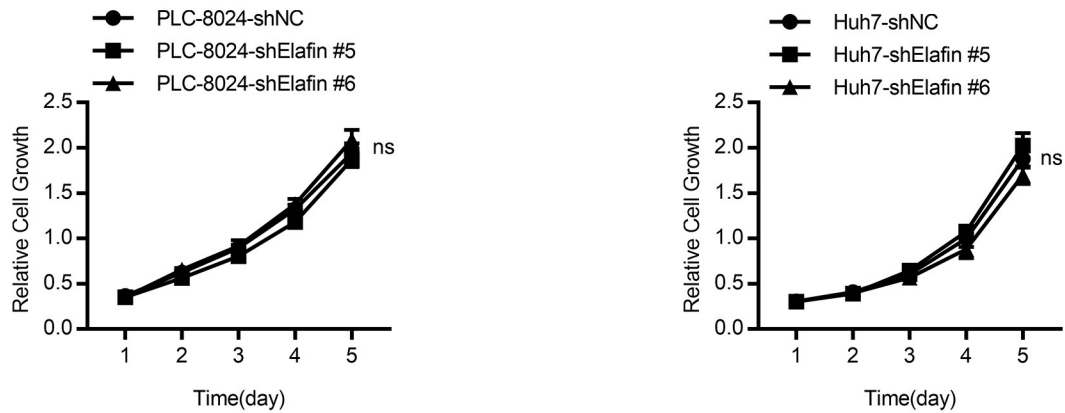

**B**

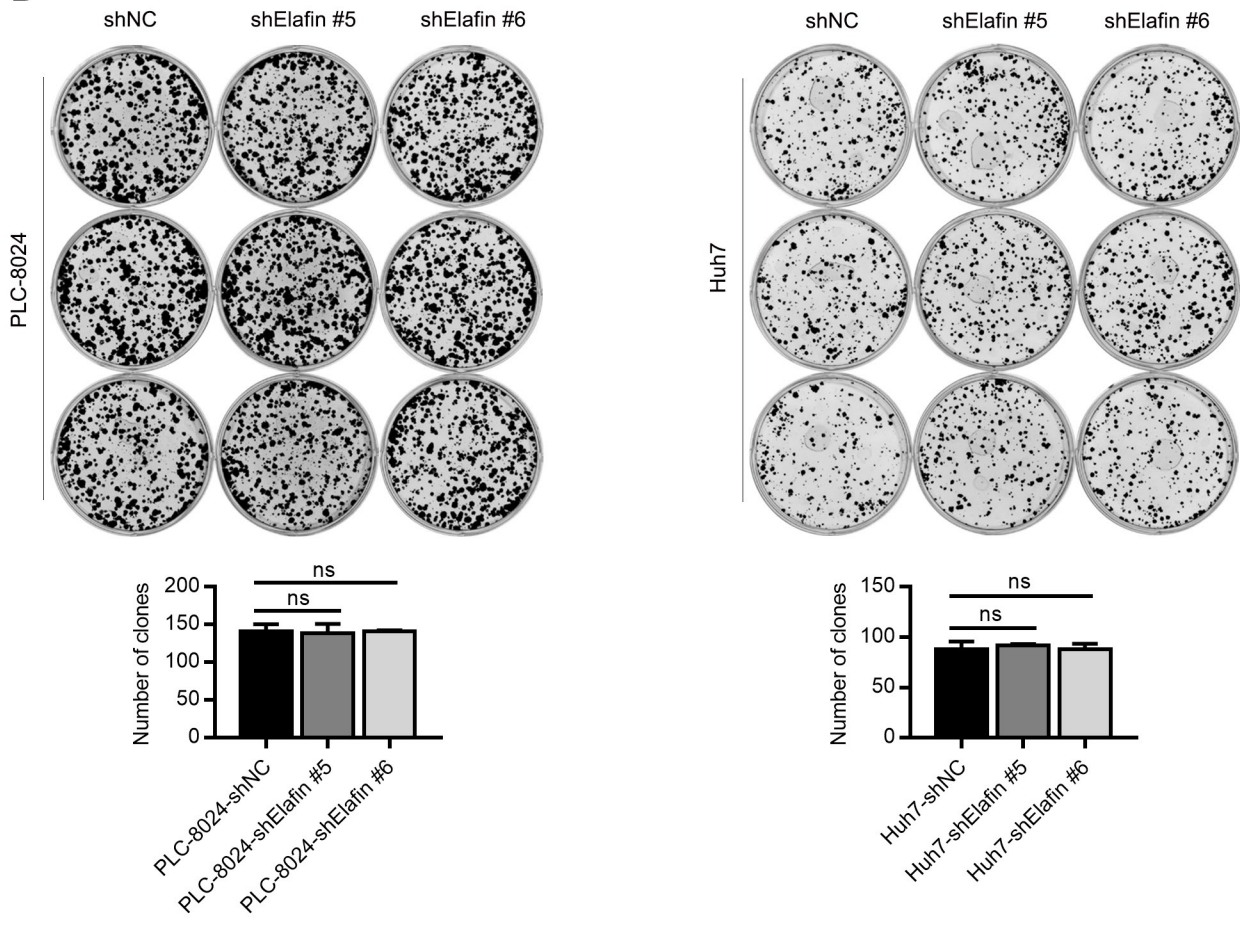

Figure S3.

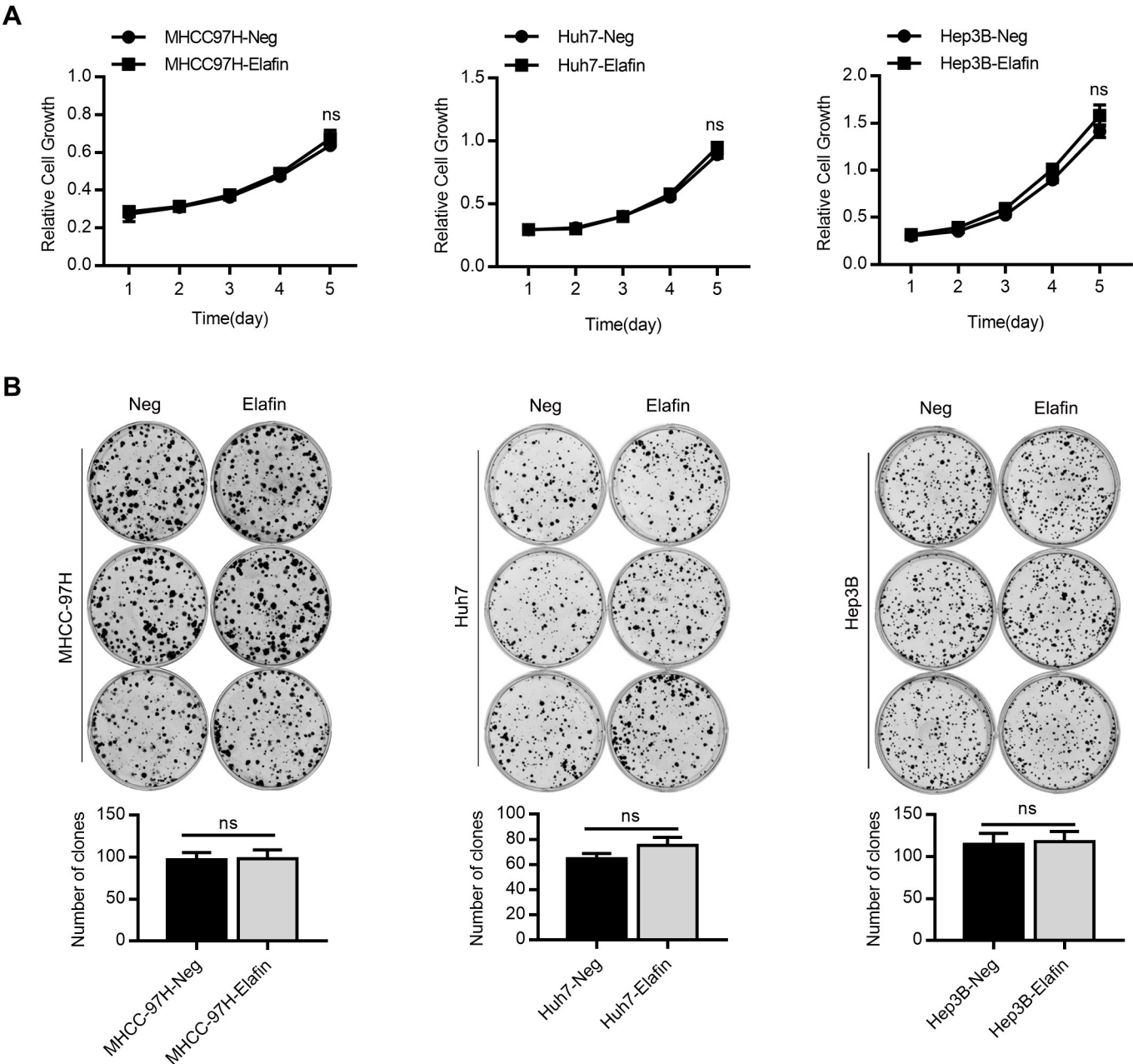

**Figure S4**

**A**

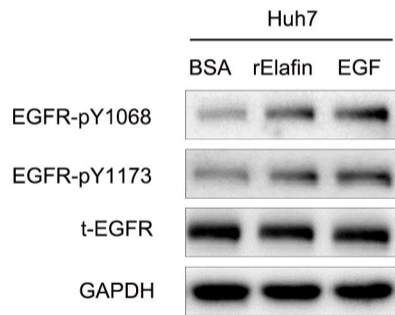

**B**

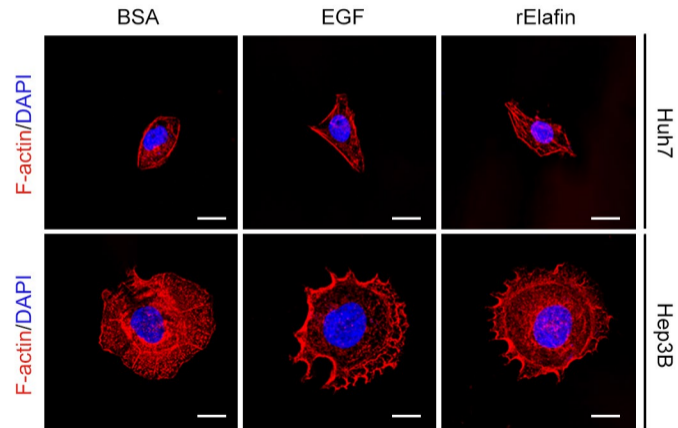

**Figure S5**

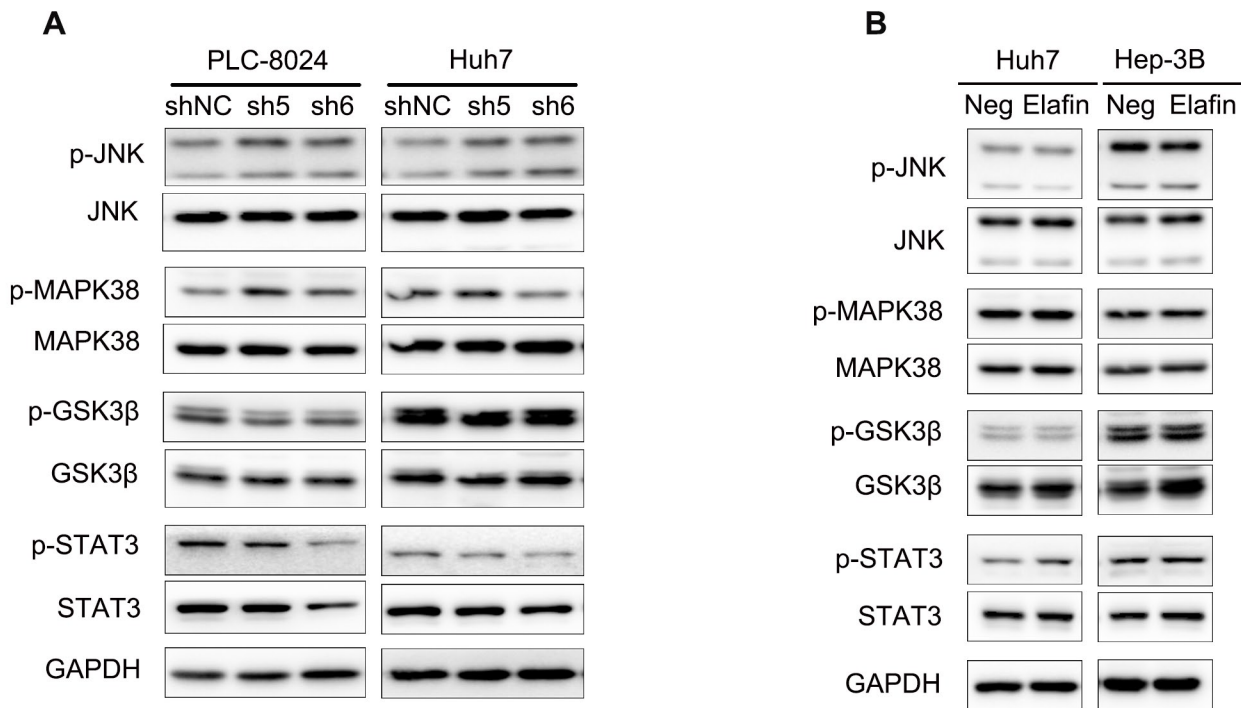

Figure S6

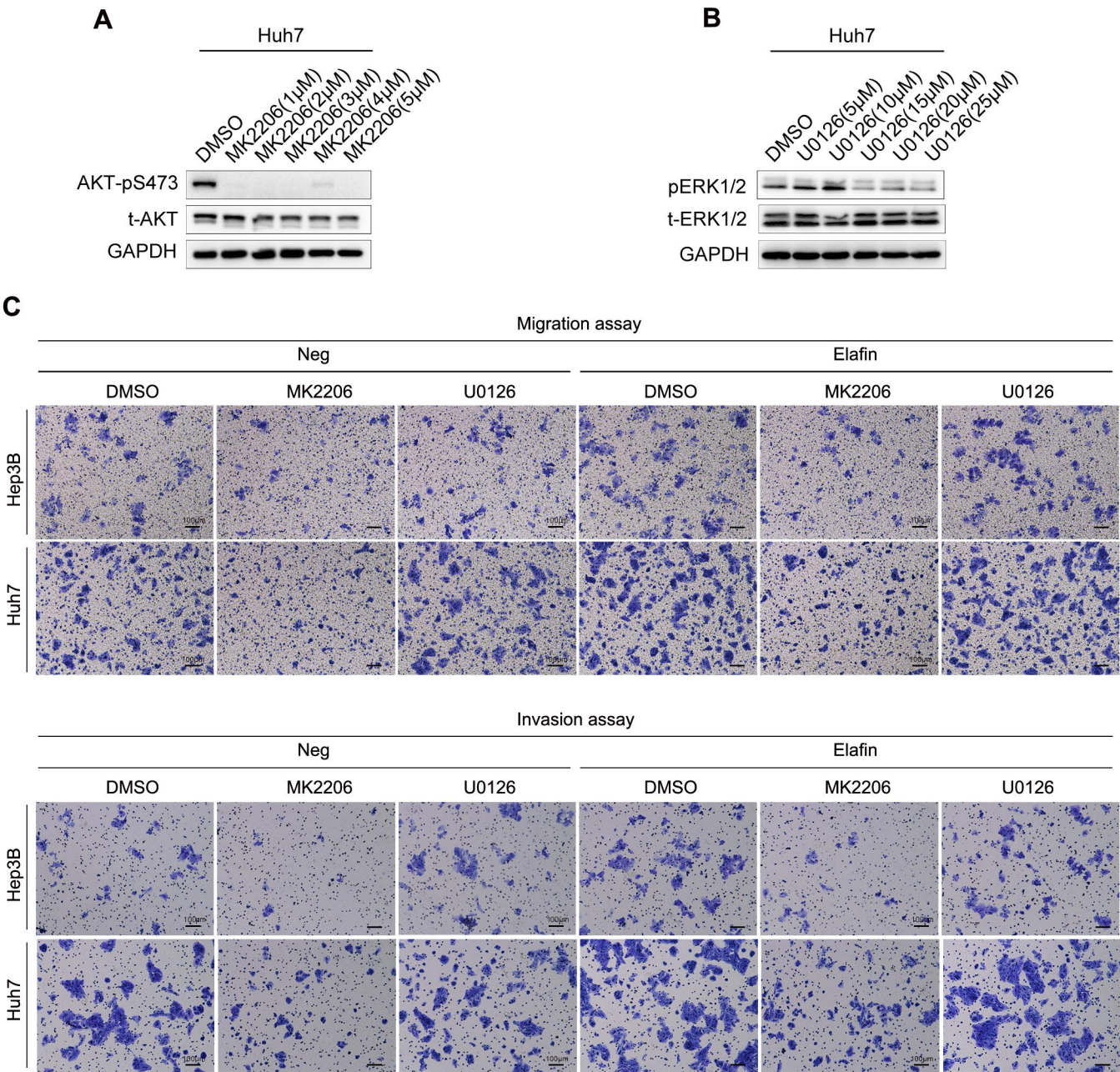

Figure S7

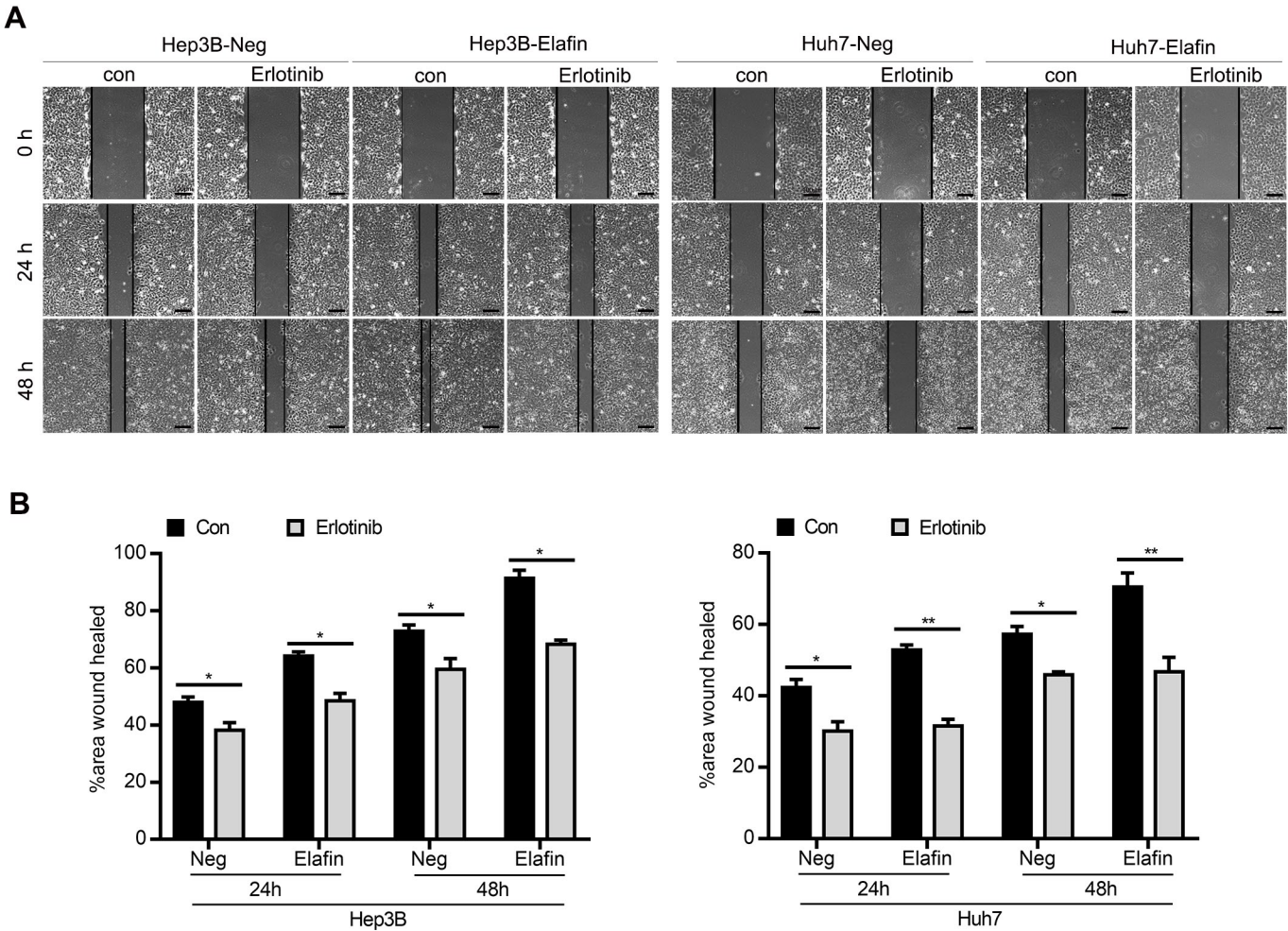

**Figure S8**

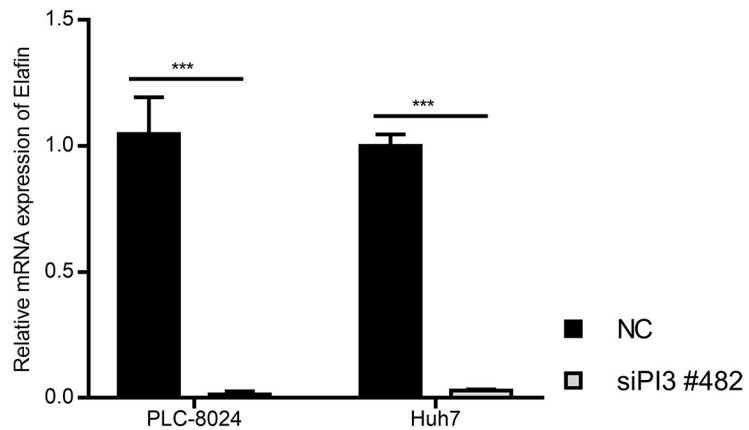

Supplement: Supplementary file 1 — Additional file 1. Supplementary Materials and Methods. [file 13046_2021_1904_MOESM1_ESM.zip › Supplementary Figures._ESM.pdf]
